# Correlation between a loss of auxin signaling and a loss of proliferation in maize antipodal cells

**DOI:** 10.3389/fpls.2015.00187

**Published:** 2015-03-26

**Authors:** Antony M. Chettoor, Matthew M. S. Evans

**Affiliations:** Department of Plant Biology, Carnegie Institution for ScienceStanford, CA USA

**Keywords:** antipodal cells, embryo sac, maize, auxin, gametophyte, Zea mays

## Abstract

The plant life cycle alternates between two genetically active generations: the diploid sporophyte and the haploid gametophyte. In angiosperms the gametophytes are sexually dimorphic and consist of only a few cells. The female gametophyte, or embryo sac, is comprised of four cell types: two synergids, an egg cell, a central cell, and a variable number of antipodal cells. In some species the antipodal cells are indistinct and fail to proliferate, so many aspects of antipodal cell function and development have been unclear. In maize and many other grasses, the antipodal cells proliferate to produce a highly distinct cluster at the chalazal end of the embryo sac that persists at the apex of the endosperm after fertilization. The antipodal cells are a site of auxin accumulation in the maize embryo sac. Analysis of different families of genes involved in auxin biosynthesis, distribution, and signaling for expression in the embryo sac demonstrates that all steps are expressed within the embryo sac. In contrast to auxin signaling, cytokinin signaling is absent in the embryo sac and instead occurs adjacent to but outside of the antipodal cells. Mutant analysis shows a correlation between a loss of auxin signaling and a loss of proliferation of the antipodal cells. The leaf polarity mutant Laxmidrib1 causes a lack of antipodal cell proliferation coupled with a loss of DR5 and PIN1a expression in the antipodal cells.

## Introduction

The plant life cycle has genetically active diploid and haploid phases, called the sporophyte and gametophyte (Walbot and Evans, 2003). The female gametophyte of angiosperms, called the embryo sac, has four cell types: the two synergids, the egg cell, the central cell, and the antipodal cells (Drews and Yadegari, 2002). The Polygonum type of embryo sac development is the most common. First, one megaspore undergoes three rounds of free nuclear divisions to produce an eight-nucleate syncytium. After the first division the two nuclei migrate to opposite poles of the embryo sac and are separated by a central vacuole. The nuclei then undergo two rounds of synchronous divisions to produce an 8-nucleate syncytium with micropylar and chalazal clusters of nuclei. The migration and position of these nuclei are highly regular. The embryo sac then cellularizes to produce seven cells. One nucleus from each pole migrates to the center of the future central cell, followed by fusion of the two nuclei in some species, and migration to the micropylar end of the central cell.

The embryo sac is polarized along the micropylar-chalazal (M-C) axis with the egg cell and synergids at the micropylar end and the antipodal cells at the chalazal end of the embryo sac. The polarization along the M-C axis is present at all stages of megagametogenesis and is present in the ovule and megaspore mother cell, thus anticipating polarity in the embryo sac. Polar distribution of cytoplasmic components along the M-C axis of the megasporocyte, a single cell, is present even before meiosis (Russell, 1979). After meiosis, the meiotic products located at the chalazal and micropylar ends can be distinguished by callose deposition and concentration of mitochondria and plastids. Only the chalazal-most of these products survives to become the functional megaspore. In the functional megaspore, a central vacuole forms during the one-nucleate stage and separates the chalazal and micropylar clusters of nuclei after the first free nuclear division. M-C polarization is apparent throughout the syncytial stages of megagametogenesis. The micropylar and chalazal poles have different concentrations of plastids and different patterns of divisions of the nuclei at each pole. Then following cellularization the cells differentiate into the cell types characteristic of their position along the M-C axis.

The antipodal cells lie at the chalazal pole of the embryo sac. Maize (*Zea mays L.*) antipodal cells are densely cytoplasmic compared to the neighboring nucellus and central cell. Maize antipodal cells can be multi- or uni-nucleate with incomplete cytokinesis so that they are only partially separated by cell walls (Diboll and Larson, 1966). The size of the antipodal cell vacuoles is also variable (Diboll, 1968). The microtubules of the antipodal cells are randomly oriented (Huang and Sheridan, 1994). They are hypothesized to function as transfer cells for the embryo sac in maize. This hypothesis for antipodal cell function is primarily based on studies of their morphology. The cell walls of maize antipodal cells adjacent to the nucellus are papillate, supporting a role for the antipodals as transfer cells for the embryo sac (Diboll, 1968). In maize, the antipodal cells continue to divide during embryo sac maturation reaching a final number of 20–100 cells with one to four nuclei each. Maize antipodal cells can persist and even continue dividing after fertilization during kernel development (Randolph, 1936). The antipodal cells of another cereal, barley, have similar cell wall invaginations juxtaposed to the surrounding nucellus and also persist beyond fertilization (Engell, 1994). Antipodal cells in maize have high sucrose synthase activity compared to the surrounding cells of the ovule, suggesting a high metabolic activity and nutritive function (Wittich and Vreugdenhil, 1998). However, the function of the antipodal cells has not been experimentally determined. Suppression of central cell identity in the antipodal cells requires the egg-cell secreted peptide ZmEAL1, indicating that egg cell signaling is critical for antipodal cell development (Krohn et al., 2012). Other factors required for antipodal cell growth and development have not yet been identified in maize.

In Arabidopsis, the antipodals do not proliferate and reportedly degenerate during embryo sac maturation, at least in starchless mutant line TL255 (Murgia et al., 1993). However, recent studies indicate that the antipodal cells of Arabidopsis persist after fertilization like those of maize, although they do not proliferate (Song et al., 2014). Interestingly, the antipodal cells of wheat also degenerate, although they proliferate first (An and You, 2004). While mutant studies have not revealed a definitive role for antipodal cells in Arabidopsis, the genetic and genomic analysis of gametophyte biology has revealed some insights into regulation of antipodal cell development. Enhancer trap and other transcriptional reporter lines have revealed that the antipodals, not surprisingly, define a unique transcriptional domain (Yu et al., 2005; Steffen et al., 2007; Bemer et al., 2010; Wang et al., 2010a; Drews et al., 2011). The neighboring central cell seems to exert influence on the development of the antipodal cells in Arabidopsis. In embryo sacs mutant for the central cell expressed *FIONA* gene, antipodal cell lifespan is increased, suggesting that a normal central cell is required to prevent persistence of the antipodals (Kagi et al., 2010). Loss of function of the chromatin cohesion factor *CTF7* also results in delayed antipodal cell death (Jiang et al., 2010). Antipodal cell specific transcripts are also actively suppressed in central cells as can be seen by the ectopic expression of antipodal cell reporters in the central cells of *agl80* and *agl61/diana* mutants (Portereiko et al., 2006; Bemer et al., 2008, 2010; Steffen et al., 2008).

Auxin is involved in many developmental processes including lateral organ development, shoot branching, and root architecture, and auxin-mediated responses depend both on patterns of auxin biosynthesis and auxin transport (reviewed in (Leyser, 2006; Zhao, 2010; Sauer et al., 2013). The main source of developmentally important auxin is a two-step tryptophan-dependent pathway (Mashiguchi et al., 2011; Phillips et al., 2011; Won et al., 2011). L-tryptophan is converted to indole-3-pyruvic acid (IPA) by *TAA1* aminotransferases (Stepanova et al., 2008; Tao et al., 2008) followed by the conversion of IPA to indole-acetic acid (IAA) by *YUCCA* (*YUC*) flavin monooxygenases (Dai et al., 2013). Control of auxin biosynthesis has been shown to be important for many environmental responses and developmental processes (reviewed in Sauer et al., 2013). Analysis of the dominant mutant *yuc1D* demonstrated that *YUCCA* flavin monooxygenases perform a rate-limiting step in auxin biosynthesis (Zhao et al., 2001). Auxin efflux under control of the PIN class of proteins is essential to achieve appropriate auxin maxima and for normal auxin signaling in a wide range of developmental contexts in Arabidopsis and maize (Mcsteen and Hake, 2001; Carraro et al., 2006; Gallavotti et al., 2008; Krecek et al., 2009; Forestan et al., 2012). Polar subcellular localization of PIN protein depends on the PINOID (PID) protein kinase and is required for normal root and shoot development (Christensen et al., 2000; Benjamins et al., 2001; Friml et al., 2004; Cheng et al., 2008). Auxin transport also depends on the ABC transporters, BRACHYTIC2 (BR2) in maize and PGP1/ABCB1 and PGP19/ABCB19 in Arabidopsis (Noh et al., 2001; Multani et al., 2003; Geisler et al., 2005) which have partially overlapping roles with PIN-dependent auxin transport (Bandyopadhyay et al., 2007; Blakeslee et al., 2007; Mravec et al., 2008). Additionally, auxin distribution is influenced by influx through AUX1 auxin influx carriers (Bennett et al., 1996; Yang et al., 2006). Auxin is perceived by the TIR1 auxin receptor, a component of an SCF-type ubiquitin protein ligase (Dharmasiri et al., 2005). Auxin binding by TIR1 leads to degradation of the AUX/IAA class of proteins; this in turn frees the AUXIN RESPONSE FACTOR (ARF) transcription factor proteins to bind DNA and modulate transcription in response to high auxin levels (for a review see, Leyser, 2006). Auxin contributes to the control of leaf polarity through *MONOPTEROS* and interactions of *ASYMMETRIC LEAVES1* (*AS1*) and *AS2* with tasiRNAs and *ETTIN/ARF3* and *ARF4* (Garcia et al., 2006; Qi et al., 2014). The maize ortholog of *AS2*, *indeterminate gametophyte1* (*ig1*), controls both leaf polarity and embryo sac development (Evans, 2007), and the dominant mutation, *Laxmidrib1-O*, has the opposite effect on leaf polarity as the recessive *ig1* mutant (Schichnes et al., 1997; Schichnes and Freeling, 1998).

Arabidopsis plants expressing GFP under the control of a *DR5* promoter reveal an auxin maximum in the micropylar nucellus during the earliest stages of embryo sac development (Pagnussat et al., 2009). Increasing auxin levels by overexpressing *YUCCA1* under control of the embryo sac promoter *pES1* disrupts embryo sac patterning with expansion of micropylar fates. Conversely, down-regulating auxin responses by expressing an artificial microRNA targeting *ARF5, ARF7*, *ARF2*, *ARF19* (and to a lesser extent *ARF8*, *ARF6*, *ARF3*, *ARF4*, and *ARF1*) blocks expression of synergid-specific (i.e., micropylar) markers (Pagnussat et al., 2009). Additional studies did not find an auxin gradient in either the Arabidopsis or maize syncytial embryo sac, and no *DR5* expression was detected in any Arabidopsis embryo sac cells (Ceccato et al., 2013; Lituiev et al., 2013). Instead auxin signaling is present in the micropylar nucellus of both species and in the antipodal cells of maize (Lituiev et al., 2013). The nucellar expression of PIN1 is required for embryo sac development in Arabidopsis (Ceccato et al., 2013). Other aspects of auxin signaling, namely AUX1 and PGP1 are localized to the plasma membrane of the female gametophyte in Arabidopsis. Here a role for auxin in the maize embryo sac is examined, including analysis of multiple gene families involved in auxin signaling and biosynthesis. Auxin signaling in maize is localized within the antipodal cell cluster, and loss of proliferation of antipodal cells is correlated with a loss of auxin signaling in the antipodal cells.

## Material and methods

### Analysis of maize gene families involved in auxin biosynthesis, distribution, and signaling

To identify maize *YUCCA*, *TAA*, *AUX1*, *brachytic2-like* ABC transporters, *PID*, and *TIR1* genes present in the maize working gene set (ZmB73 v.5a.59), the Working Gene Set Peptide database was queried using BLAST at http://maizegdb.org starting with the published Arabidopsis *YUCCA1*, *TAA1*, *AUX1*, *PID* (and maize *bif2*) and *TIR1* genes and their close homologs. To ensure that related *bona fide* maize orthologs of *YUCCA1* could be distinguished from other classes of monooxygenases, *FMO1* and related flavin monooxygenases of Arabidopsis (Bartsch et al., 2006) were included in the phylogenetic analysis of the *YUCCA1* family. Similarly, the Jasmonate receptor, *COI1* (Thines et al., 2007), was used as an outgroup for the *TIR1* auxin receptor family, and the amino acid transporter AT5G41800 as an outgroup for the *AUX1* family of auxin influx carriers. Maize *ARF* transcription factor nomenclature is based on published results (Xing et al., 2011) and the Grass Transcription Factor Database (http://grassius.org/tf_browsefamily.html?species=Maize) (Yilmaz et al., 2009). Only *ARF* genes with a full-length B3 domain were included in the analysis. The list of maize *IAA* genes was taken from the annotated gene set at maizesequence.org (http://www.maizesequence.org), which includes the published *IAA* gene list (Wang et al., 2010b) with some modifications to the family members caused by the update of the ZmB73 genome from version 4a.53 to 5a.59. The maize *PIN* gene family nomenclature was taken from published analysis (Forestan et al., 2012) with one additional gene identified by BLAST query of the maize genome (ZmB73 v.5a.59). For the maize PID/BIF2 protein kinase family, analysis focused on genes in the PID/PID2/WAG1/WAG2 clade because these genes are functionally redundant for auxin control of cotyledon development (Cheng et al., 2008), although phylogenetic analysis included a larger group of serine-threonine kinases. For all phylogenetic analyses, alignments were made using the ClustalW algorithm in MegAlign (DNASTAR). Phylogenies were produced from these alignments using MrBayes v3.2.0 using default settings for amino acid analysis (Huelsenbeck and Ronquist, 2001). Each MrBayes analysis was performed for 100,000 generations or until the standard deviation of the split frequencies dropped below 0.05. The *PIN1* family analysis was run for 200,000 generations. The *ARF* family was run for 820,000 generations. The *AUX1*, *PID/WAG*, *YUCCA*, *TAA1*, *TIR1*, and ABC transporter family analyses were run for 100,000 generations each. Phylogenetic trees were drawn from the MrBayes files using FigTree v1.4.0 (http://tree.bio.ed.ac.uk/software/figtree/). Gene expression values were taken from RNA-Seq data from Illumina sequencing of B73 mature, freshly shed pollen and B73 5-day old seedling shoot, and combined RNA-Seq data from Illumina sequencing of B73 embryo-sac-enriched samples and ovules with the embryo sacs removed and from SOLiD sequencing of W23 embryo-sac-enriched samples and ovules with the embryo sacs removed (Chettoor et al., 2014). This data was mined for expression levels based on Fragments per Kilobase per Million reads (FPKM) for the genes in the gene families above. Genes were considered up-regulated in the embryo-sac-enriched samples if they were 2-fold higher than the surrounding ovule tissue with an expression threshold above 0.1 FPKM.

### Microscopy of embryo sacs

Analysis of fixed embryo sacs by confocal microscopy was performed without additional staining after FAA fixation according to Phillips and Evans (2011) or alternatively after staining with Acriflavine alone or with both Acriflavine and Propidium Iodide. Tissues were stained with Acriflavine as a Schiff reagent as published previously (Vollbrecht and Hake, 1995) and some samples, after Acriflavine staining and before dehydration in ethanol and clearing in Methyl Salicylate, were stained with Propidium Iodide (Running et al., 1995). Acriflavine/Propidium Iodide stained samples were visualized on a Leica Sp5 point-scanning confocal microscope using excitations of 436 and 536 nm and emissions of 540 ± 20 and 640 ± 20 nm. For live cell imaging of fluorescent reporters in maize ovules, dissection of the ovules was performed similarly but without fixation from plants carrying one copy of either the *pHISTONE H1B(GRMZM2G164020)::HISTONE H1B-YFP*, *pDR5::RFP*, *pPIN1(GRMZM2G098643*)*::PIN1-YFP*, or *pTCS::TCSv2::NLS-tdTomato* transgene in a B73 inbred background. All transgenic lines were generously supplied by the Maize Cell Genomics Project (http://maize.jcvi.org/cellgenomics/index.php) (Mohanty et al., 2009). To dissect out ovules for fluorescence microscopy, freshly harvested ears were kept in a humid environment with the husks only partially removed and only a few ovules dissected at a time. The silk was removed to the base of the silk to expose the ovule. The ovule was then bisected along the longitudinal axis of the ear to produce a cut surface within a few cell layers of the embryo sac. The cut surfaces were then placed against a cover slip in water for observation on an inverted microscope with a Leica SP5 point-scanning confocal microscope using an excitation of 514 nm and an emission of 550 ± 20 nm for YFP, an excitation of 563 nm and an emission of 600 ± 20 nm for RFP, and an excitation of 554 nm and an emission of 600 ± 20 nm for tdTomato. For analysis of effects of *Lxm1-O* on expression of fluorescent reporters *Lxm1-O/+* plants were crossed as males to transgenic hemizygotes. Live cell imaging of ovules of plants hemizygous for the transgene and heterozygous for *Lxm1-O* were examined in the same way as wild type. Ovule/embryo sac staging was performed using silk length as a proxy for ovule age similarly to Huang and Sheridan (1994). Ovules of florets with silks over 15 cm in length were taken as mature stage, with shorter silks an estimate of progressively younger ovules.

## Results

### Maize antipodal cells

**Figure 1:**
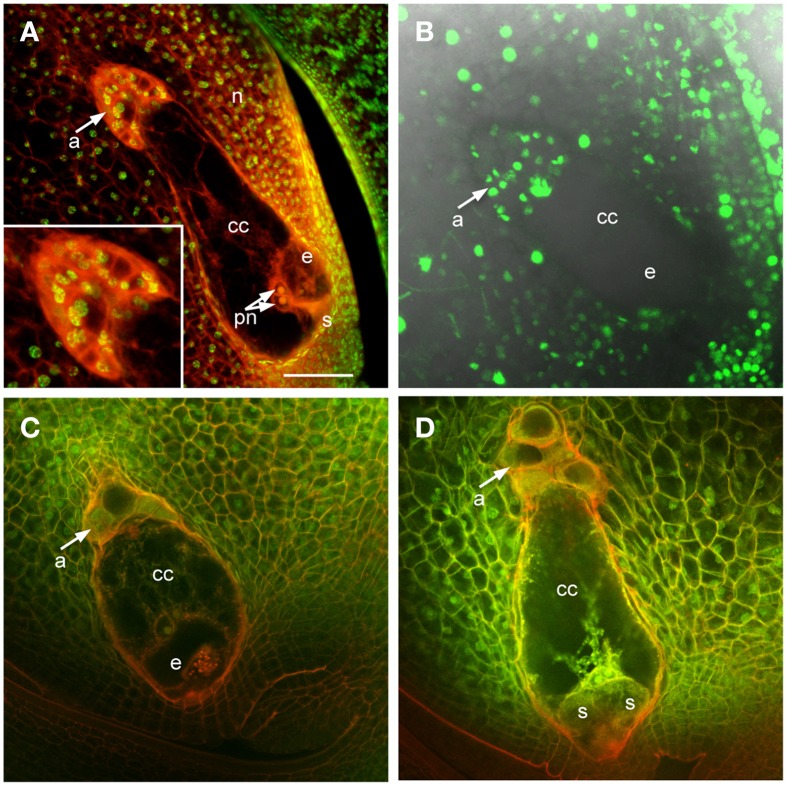
**Maize ovules with mature embryo sacs. (A)** Fixed and cleared ovule stained with Acriflavine (Red) and Propidium Iodide (Green). Inset shows higher magnification of antipodal cell region. **(B)** Live cell imaging of an ovule expressing *pHistoneH1B::HISTONEH1B-YFP*. **(C,D)** Fixed and cleared ovules stained with Acriflavine (Red) and fluorescence from formaldehyde fixation and autofluorescence (Green). a, antipodal cells; cc, central cell; e, egg cell; n, nucellus; pn, polar nuclei; s, synergid. Scale bar = 100 μm.

In maize and other grasses the antipodal cells are unique among embryo sac cells in proliferating after cellularization. This is one of the distinguishing features of grass embryo sacs compared to Arabidopsis, in which the antipodal cells do not proliferate. Analysis of mature embryo sacs reveals that the mature antipodal cells also have fundamental differences from other embryo sac cells. Nuclei of the antipodal cells are very distinct from those of the central cell or egg cell (the synergids have typically degenerated by maturity) and more closely resemble the surrounding nucellar cell nuclei (Figure 1). Egg cell nuclei and particularly polar nuclei have large prominent nucleoli, which stain with Acriflavine as a Periodic Acid Schiff reagent, and the nuclei overall stain faintly with Propidium Iodide. The antipodal cell nuclei, in contrast, lack prominent nucleoli and have an intense speckled staining pattern with Propidium Iodide. Additionally, the Histone H1B gene, GRMZM2G164020, is expressed in the antipodal cells and the nucellus, but not in mature central cells or egg cells (Figure 1B). Based on nuclear staining properties, nucleoli appearance, and Histone H1B expression, the antipodal cell nuclei are much more similar to the nucellar nuclei than they are to the other embryo sac nuclei.

Early after cellularization, most or all of the antipodal cells are cytoplasmically dense and fluoresce intensely with FAA fixation (Figures 1C,D). The size and fluorescent properties of the antipodal cells vary with inbred background (data not shown). The boundary between the antipodal cell cluster and the nucellus stains intensely with Acriflavine as a Schiff reagent. The most chalazal of the antipodal cells is often more vacuolated than the rest in early stages of antipodal cell cluster development; as the antipodal cell cluster grows and matures more of them become vacuolated.

### Auxin signaling in maize antipodal cells

**Figure 2:**
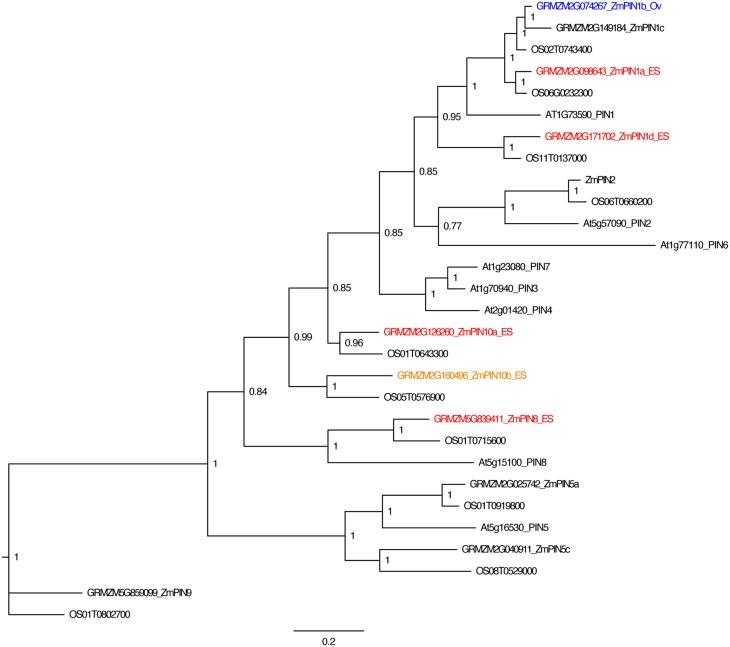
**PIN gene family of maize**. Phylogenetic relationships of maize and Arabidopsis PIN genes. Maize PIN genes up-regulated two-fold in the embryo-sac-enriched samples (and over 0.1 FPKM) compared to the surrounding ovule tissue are indicated in red, while genes with higher expression in the surrounding ovule tissue than the embryo sac are indicated in blue. Genes indicated in orange have higher expression in the embryo sac than the surrounding ovule but either fall below the 0.1 FPKM cutoff or are only 1.5 to 2.0 fold higher in the embryo sac compared to the ovule. Gene names are according to Forestan et al. (2012).

To analyze the pattern of auxin signaling in the maize embryo sac, the expression pattern of two fluorescent reporters in maize were studied: a transcriptional reporter of auxin levels, *DR5::RFP*, and a fluorescent protein fusion for a auxin efflux carrier (GRMZM2G098643_ZmPIN1a) expressed from its native promoter, *pPIN1a::PIN1a-YFP* (Gallavotti et al., 2008). Maize whole embryo sac RNA-Seq data was mined to determine whether this *PIN* gene is likely to be expressed in the embryo sac. RNA-Seq of embryo-sac-enriched samples (with some attached nucellus) was compared to the remainder of the ovule lacking the embryo sac (Chettoor et al., 2014). *ZmPIN1a*, along with three other maize *PIN* genes, is up-regulated in the embryo sac (defined as having 2-fold higher expression in the embryo sac enriched sample compared to the surrounding ovule tissue and expression above 0.1FPKM) (Table 1 and Table S1). These four maize *PIN* genes fall into three different groups, 1, 10, and 8 (using the nomenclature of Forestan et al., 2012) (Figure 2). Two additional genes are two-fold higher in the embryo sac compared to the surrounding ovule but have expression below the 0.1 FPKM threshhold. One gene, GRMZM2G074267 in the clade with *ZmPIN1a* and *AtPIN1*, has the reverse expression pattern with higher expression in the surrounding ovule than the embryo sac.

**Table 1:** **Expression of gene families related to auxin movement, signaling, and biosynthesis in maize embryo sacs**.

**Gene Family**	**Genes up-regulated in the embryo sac compared to surrounding ovule tissue**	
	**2-fold Higher in ES vs. surrounding ovule and above 0.1 FPKM**	**1.5-fold higher in ES vs. surrounding ovule and above 0.05 FPKM**
PIN	4 of 10	5 of 10
AUX1	1 of 5	3 of 5
BR2-like	3 of 8	4 of 8
PID	2 of 5	2 of 5
ARF	9 of 35	11 of 35
AUX/IAA	16 of 37^†^	23 of 37^†^
TIR1-like	0 of 8	2 of 8
YUCCA	5 of 11	5 of 11
TAA	4 of 6^*^	5 of 6^†^
All auxin-related families	44 of 125^†^	60 of 125^†^
Whole genome	9618 of 39,635 (3414 in surrounding ovule)	13,579 of 39,635 (6354 in surrounding ovule)

† Higher than expected based on whole genome frequency of ES up-regulated genes, p < 0.01.

* Higher than expected based on whole genome frequency of ES up-regulated genes, p < 0.05.

**Figure 3:**
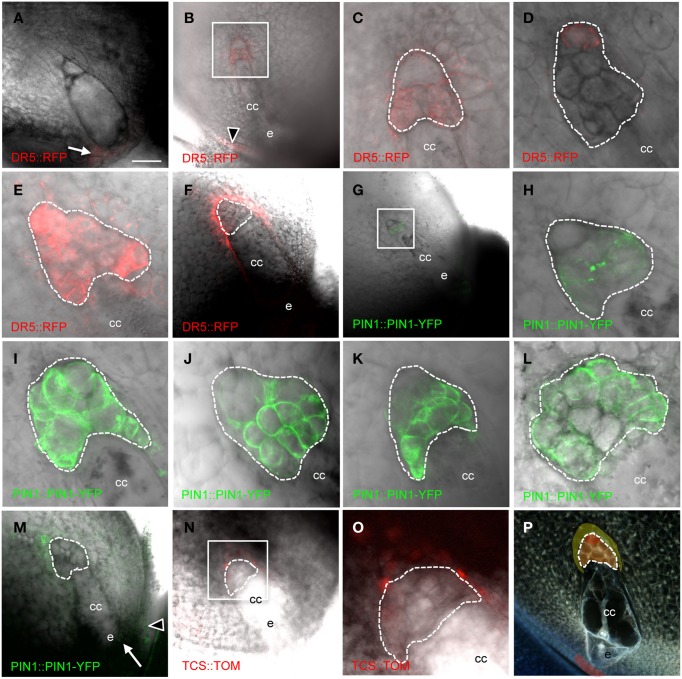
**Cellularized maize embryo sacs showing expression in the antipodal cells of (A–F) DR5::RFP reporter, (G–M) PIN1a::PIN1a-YFP reporter, and (N–O) TCS::TOMATO reporter**. The chalazal tip of the antipodal cell cluster is oriented toward the upper left. **(A)** Embryo sac just prior to cellularization. **(B,C,G,H)** Early post-cellularized embryo sac with 6–10 antipodal cells. **(D–F;I–P)** Mature embryo sacs. **(B–E)** DR5::RFP expression in the antipodal cells. **(F)** DR5::RFP expression in the sporophytic tissues of the nucellus. **(C)** Boxed region in **(B)**. **(H)** Boxed region in **(G)**. **(O)** Boxed region in **(N)**. **(P)** Model for auxin and cytokinin signaling in the mature maize embryo sac. Red indicates region of highest auxin signaling; orange indicates moderate auxin signaling; and yellow indicates low auxin signaling plus cytokinin signaling. Dashed lines indicate boundary of antipodal cells. cc, central cell; e, egg cell. Arrow indicates micropylar nucellus, and arrowhead indicates integuments. Scale bar = 100 μm **(A,B,F,G,M,N,P)** and = 33 μm **(C–E,H–L,O)**.

*PIN1a* has complex patterns in the antipodal cell cluster of mature maize embryo sacs (Figure 3 and Table 2). PIN1a-YFP is detectable early in antipodal cell development at least as early as the 6–10 cell stage in all but the most chalazal antipodal cells (Figures 3G,H). In later stages, the most common patterns of PIN1a-YFP expression are: expression throughout the antipodal cell cluster (Figure 3I), expression in all cells of the antipodal cell cluster except the most chalazal cell (Figure 3J), and expression in the micropylar portion of the antipodal cell cluster with multiple cells at the chalazal end lacking (or with reduced) expression of PIN1a-YFP (Figure 3K). Less frequently, PIN1a-YFP protein is expressed in all but the micropylar domain of antipodal cells or all but the center of the antipodal cell cluster. The least frequent pattern has PIN1a-YFP expression only in the center of the antipodal cell cluster. Using the positions of the cell walls with the strongest expression of PIN1a-YFP as a proxy for the direction of auxin flow the two most common patterns are outward from the antipodal cell cluster and away from the central cell or random within the antipodal cell cluster. These patterns suggest that the auxin efflux pattern is dynamic but within the antipodal cell cluster. Whether these patterns represent different stages in mature or nearly mature antipodal cell clusters is difficult to determine without being able to maintain their growth *in vitro*.

**Table 2:** **Patterns of expression of *pPIN1::PIN1-YFP* in mature antipodal cell clusters**.

**All antipodal cells**	**Expression absent (or Reduced) in the Chalazal-most antipodal cell**	**Expression absent in multiple antipodal cells at the Chalazal end**	**Expression absent in the micropylar domain of the antipodal cell cluster**	**Expression absent in the center of the antipodal cell cluster**	**Expression only in the center of the antipodal cell cluster**
35	34	31	8	11	2

DR5 expression is also detected early after cellularization, at least as early as the 6-10 cell stage in all cells except the most chalazal antipodal cell (Figures 3B,C). At maturity, maximal expression of *DR5* in the embryo sac is detected in the chalazal-most cell of the antipodal cell cluster (Figure 3D). Interestingly, this is the same cell that often has the lowest expression of PIN1a-YFP. Increasing the sensitivity for detection of lower levels of *DR5*-driven RFP expression reveals that DR5 expression is higher in all of the antipodal cells than the surrounding nucellar cells (Figure 3E). Since the *pDR5::RFP* construct is hemizygous, half of the embryo sacs do not carry the transgene and consequently do not express *RFP*. Without the interfering antipodal cell fluorescence in these ovules, it was possible to reveal DR5 expression in the nucellus at a lower level than any of the antipodal cells. Nucellar *DR5* expression is located in the cells immediately adjacent to the chalazal end of the embryo sac surrounding the antipodal cells (Figure 3F). Similarly, in the ovules of *pPIN1a::pPIN1a-YFP* hemizygotes in which the embryo sac did not inherit the transgene, low *PIN1a* expression could be detected in the nucellus adjacent to the antipodal cells (Figure 3M). Elsewhere in the ovule, expression of *DR5* and *PIN1a-YFP* is seen in the integuments and the micropylar nucellus between the embryo sac and the micropyle (Figures 3A,B,M).

One model for regulation of embryo sac development is an antagonistic relationship between auxin and cytokinin. Interplay between auxin and cytokinin are involved in other processes (Moubayidin et al., 2009), including an inverse correlation between expression of DR5 and the cytokinin-responsive TCS promoter in the embryo (Muller and Sheen, 2008). To test if cytokinin signaling antagonizes auxin in the embryo sac the *TCS* promoter was examined to determine if there is an inverse correlation between DR5 and TCS in the embryo sac. While no expression from the *TCS* promoter was detectable within the embryo sac, the nucellar cells immediately chalazal to the antipodal cells express the *TCS* reporter (Figures 3N,O). These are the same cells that have low-level *DR5* expression (i.e., below the level of the antipodal cells but above that of other nucellar cells).

*DR5* expression reveals that transcriptional responses to auxin are active in the antipodal cells presumably through the release of ARF transcription factors from AUX/IAA proteins in the presence of high auxin. To identify the endogenous transcriptional targets of auxin in the embryo sac it is necessary to determine which ARFs are expressed in these cells to control transcriptional changes in response to auxin. Additionally, to determine if other proteins involved in auxin transport and the local synthesis of auxin contribute to the pattern of auxin responses, transcriptome data from embryo-sac-enriched (ES) tissue samples and from the surrounding ovule tissue without embryo sacs (Ov) were mined for multiple components of auxin signaling and biosynthesis (Table 1 and Tables S2–S9).

The set of auxin related genes is over-represented in the ES up-regulated gene set compared either to the whole genome or to Ov up-regulated genes (Table 1). In addition to the PIN proteins, the *AUX1*-like auxin influx carriers, *BR2*-like ABC transporter families, and *PINOID* type protein kinases have members with higher expression in the embryo-sac-enriched tissue than the surrounding ovule suggesting these pathways are operating in the maize embryo sac (Tables S2–S4, Figures S1–S3). In contrast to Arabidopsis which expresses *AUX1* but not *LAX1, 2*, or *3* in the embryo sac (Lituiev et al., 2013) maize embryo sacs express genes in the *LAX2/LAX3* half of this family (Figure S1). Auxin distribution depends on the localization of auxin biosynthesis as well as transport (Zhao et al., 2001). Members of the gene families for both steps of auxin biosynthesis—by TAA1 aminotransferases and YUCCA flavin monooxygenases —are up-regulated in the embryo sac compared to the surrounding ovule (Tables S5,S6; Figures S4,S5). Therefore, it is likely that the auxin that accumulates in the antipodal cells is synthesized locally, either in or adjacent to the embryo sac. The *YUCCA* genes up-regulated in the embryo sac fall into the *YUCCA10* and *YUCCA2/6* branches (Figure S5).

**Figure 4:**
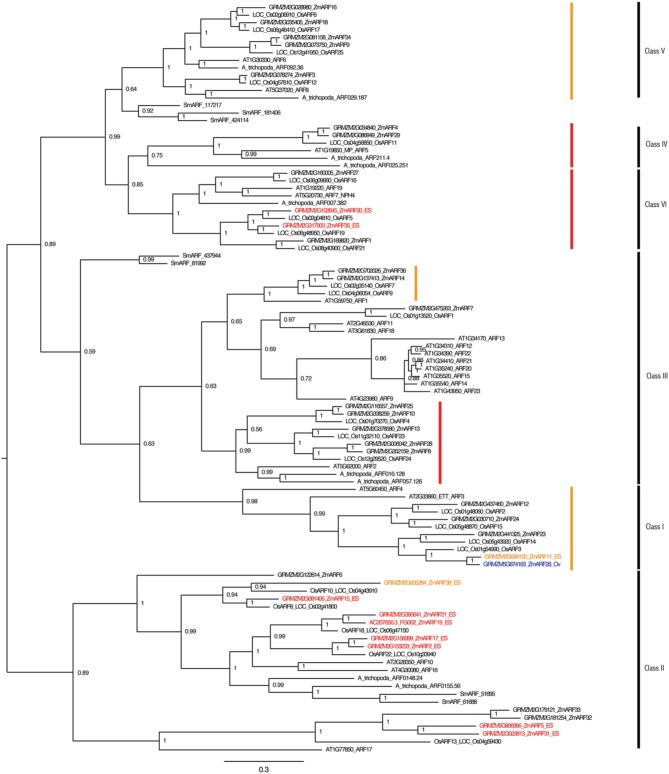
**ARF gene family of maize and Arabidopsis**. Maize genes up-regulated two-fold in the embryo-sac-enriched samples (and over 0.1 FPKM) compared to the surrounding ovule tissue are indicated in red, while the genes with higher expression in the surrounding ovule tissue than the embryo sac are indicated in blue. Genes indicated in orange have higher expression in the embryo sac than the surrounding ovule but either fall below the 0.1 FPKM cutoff or are only 1.5 to 2.0 fold higher in the embryo sac compared to the ovule. Family classes are given according to Xing et al. (2011). Branches with family members whose downregulation in Arabidopsis by an artificial microRNA (Pagnussat et al., 2009) caused abnormal embryo sac development are marked by red (strongly targeted) or orange (more weakly targeted) bars.

Gene families involved in auxin perception and response were also examined for embryo sac expression. Representatives of the *TIR1*, *ARF*, and *IAA* gene families have higher expression in the embryo-sac-enriched samples than the surrounding ovule (Tables S7–S9; Figure 4, Figures S6,S7). RNA-seq analysis revealed that nine of the thirty-five *ARF* genes are expressed 2-fold higher in the embryo-sac-enriched samples than the surrounding ovule tissue plus two more with a weaker increase in the embryo-sac-enriched samples (Table S8). The Class II *ARF* group is over-represented among ES up-regulated genes; seven of the eleven Class II genes have higher expression in the embryo sac enriched samples, while only two of the remaining twenty-four *ARF* genes do, both of which are in Class VI (Figure 4). The function of this clade in embryo sac development is unknown. In Arabidopsis, combined down-regulation of several *ARF* genes caused abnormal embryo sac development (Pagnussat et al., 2009), but the artificial microRNA targeting *ARFs* in this study did not cover the Class II group, which includes most of the genes with increased expression in the maize embryo-sac-enriched samples.

### *Laxmidrib1* mutants interfere with proliferation and auxin signaling in the antipodal cell cluster

**Figure 5:**
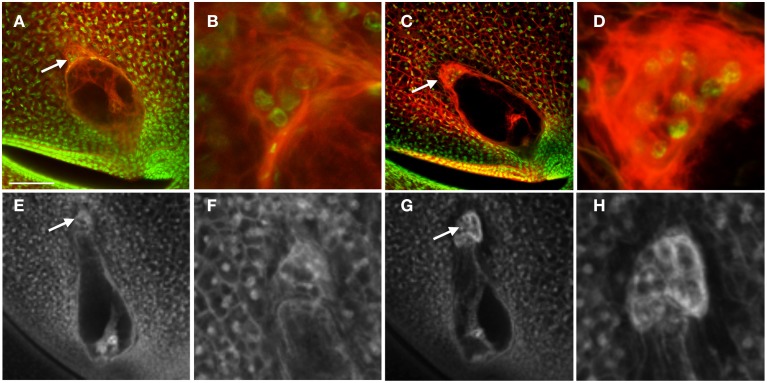
**Effect of *Lxm* mutations on embryo sac development. (A–D)** Embryo sacs from a *Lxm1-O/+*; W23 heterozygote fixed in FAA and stained with Acriflavine and Propidium Iodide and **(E–H)** Embryo sacs from a Lxm^*^-N2530 heterozygote in a hybrid genetic background fixed in FAA. **(A,B,E,F)** Embryo sacs with abnormal antipodal cell clusters. **(C,D,G,H)** Normal sibling embryo sacs for each mutant line. **(B,D,F,H)** are magnifications of the antipodal cells in **(A,C,E,G)**, respectively. Arrows indicate antipodal cell cluster. Scale bar = 100 μm **(A,C,E,G)** and = 33 μm **(B,D,F,H)**.

To determine a potential function for the auxin maximum in the antipodal cells, we then examined the effect of an antipodal cell mutant on the expression of the *DR5* and *PIN1* fluorescent reporters. Because the *indeterminate gametophyte1* (*ig1*) mutation affects both leaf polarity and embryo sac development (Evans, 2007), other leaf polarity mutants were examined for effects on embryo sac morphology. The *Laxmidrib1-O* (*Lxm1-O*) mutant is a dominant mutant with adaxialized leaves. Sectors of adaxial tissue are produced on the abaxial side of leaves with ectopic leaf flaps produced on either side of these sectors (the opposite leaf polarity defect as *ig1*) (Schichnes et al., 1997; Schichnes and Freeling, 1998). Approximately one third of the embryo sacs in *Lxm1-O/+* heterozygotes in a W23 inbred background are abnormal (39/122) (Figure 5). These embryo sacs have fewer antipodal cells than their wild-type siblings (six antipodal cell nuclei in Figures 5A,B), indicating that proliferation of the antipodal cells in mutant embryo sacs is reduced or in some cases absent. The size and morphology of these antipodal cells are similar to wild type, however, and, like wild type, the nuclei have speckled staining with Propidium Iodide and lack prominent nucleoli. The overall size and morphology of the central cell, egg cell, and synergids are not affected by *Lxm1-O*. A second mutation, Lxm^*^-N2530, which has similar effects on leaf development as *Lxm1-O*, also produces embryo sacs with smaller antipodal cell clusters than wild type, supporting the argument that this is a result of the *Lxm* mutations rather than a second mutation segregating in the background (Figures 5E–H). The normal and mutant antipodal cells of the Lxm^*^-N2530 line are slightly larger and more vacuolated than those of the *Lxm1-O* line, but these mutations are in different inbred backgrounds.

**Figure 6:**
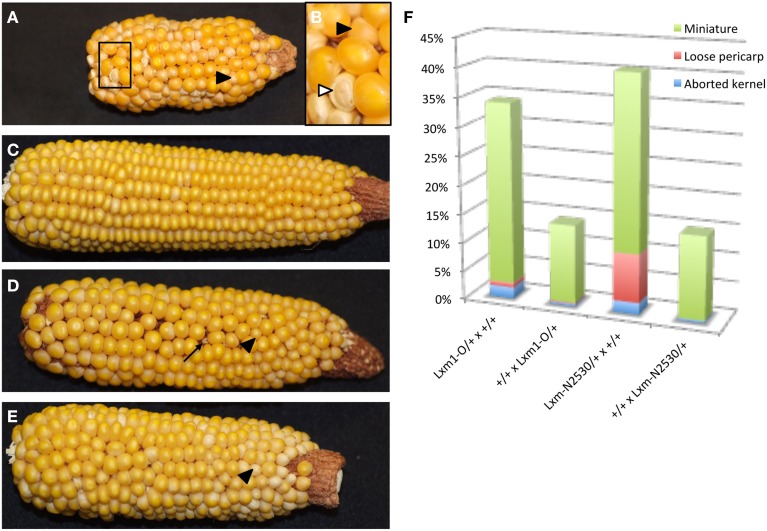
**Reciprocal crosses between *Lxm1/+* and wild-type siblings**. Black arrowheads point to miniature kernels. **(A)** *Lxm1/+* female with mild leaf phenotype crossed by wild-type male. **(B)** Enlargement of boxed region in **(A)**. White arrowhead points to a kernel with a loose pericarp. **(C)** Wild-type female crossed by *Lxm1/+* male with mild leaf phenotype. **(D)** *Lxm1/+* female with strong leaf phenotype crossed by wild-type male. Arrow points to an aborted kernel. **(E)** Wild-type female crossed by *Lxm1/+* male with strong leaf phenotype. **(F)** Frequency of abnormal kernel types in reciprocal crosses between *Lxm1-O* or *Lxm-N2530* heterozygotes and homozygous wild type. Female genotypes are listed first.

Heterozygotes for both *Lxm* mutations also produce miniature seeds of different severity and frequency depending upon the direction of the cross (Figure 6). *Lxm1-O/+* and Lxm^*^-N2530/+ females segregate kernels that are small and pale with a loose pericarp. Progeny testing of kernels from crosses of *Lxm/+* females by homozygous wild-type males revealed that inheritance of the mutation (i.e., the fertilization of mutant embryo sacs) is correlated with the miniature kernel phenotype (19/21 miniatures that were tested had inherited *Lxm* but only 2/31 normal kernels tested had inherited *Lxm*). Although the crosses of *Lxm/+* males onto wild type do not produce the reduced endosperm, loose pericarp class of kernels, some crosses do produce a less severe miniature kernel type (Figure 6E), particularly in crosses using *Lxm* plants with the most severe leaf phenotype. When crossed as females, these severe *Lxm* heterozygotes produce some miniatures and some early aborting kernels and have partial sterility (Figure 6D). All abnormal kernel types are more common in crosses with *Lxm* females than males, especially the most severe classes (Figure 6F). The loose pericarp and aborted kernel phenotypes are rarely seen in crosses with *Lxm1-O* or Lxm^*^-N2530 males but are found in females of both mutants.

**Figure 7:**
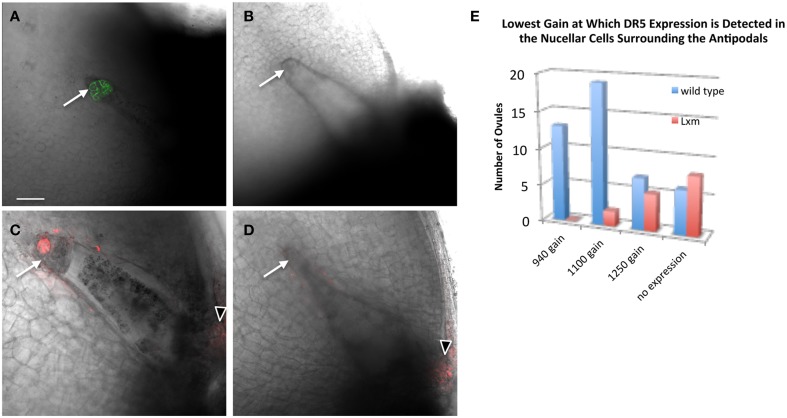
**Effect of *Lxm1-O* on embryo sac expression of *pPIN1::PIN1-YFP* and *pDR5::RFP***. Live cell imaging of sibling **(A,C)** wild-type and **(B,D)** *Lxm1-O* embryo sacs from *Lxm1-O* heterozygotes segregating either **(A,B)** *pPIN1::PIN1-YFP* or **(C,D)** *pDR5::RFP*. **(A)** Embryo sac expressing *pPIN1::PIN1-YFP*. **(C)** Embryo sac expressing *pDR5::RFP*. The small antipodal cell cluster distinguishes *Lxm1-O* from wild-type. Neither *DR5* nor *PIN1* are expressed in mutant *Lxm1-O* embryo sacs. Arrows indicate antipodal cell cluster. Arrowheads indicate DR5 expression in the micropylar nucellus. Scale bar = 100 μm. **(E)** Embryo sacs were visualized at three different sensitivity settings for *DR5* expression: a gain of 940, 1100, or 1250. This was used as a proxy for relative *DR5* expression between embryo sacs. For most of the normal embryo sacs, *DR5* expression could first be detected at the lower gain settings used, while for most of the mutant embryo sacs *DR5* expression either was not detected at all or was only detected at the highest setting.

To test whether *Lxm1-O* affects auxin distribution in the embryo sac, *pPIN1a::PIN1a-YFP* and *pDR5::RFP* were crossed with *Lxm1-O*/+ mutant lines. Plants heterozygous for *Lxm1-O* and hemizygous for either *pPIN1a::PIN1a-YFP* or *pDR5::RFP* were examined for effects of *Lxm1-O* on auxin signaling in the embryo sac. Examination of mutant plants revealed that the *Lxm1-O* mutation interferes with expression of both *PIN1a* and *DR5* in maize antipodal cells (Figure 7 and Table 3). In plants heterozygous for *Lxm1-O* and hemizygous for *pPIN1a::PIN1a-YFP*, approximately half of the normal embryo sacs express the transgene, as expected, but none of the abnormal embryo sacs express the transgene. The effect of *Lxm1-O* on *pDR5::RFP* expression is essentially the same as for *pPIN1a::PIN1a-YFP*, with only 1 of 17 abnormal embryo sacs expressing the transgene, with the one exceptional individual having an intermediate antipodal cell phenotype. For *pDR5::RFP* fewer than half of the wild type embryo sacs express the transgene, perhaps reflecting silencing of this construct, but still a significantly higher frequency of normal embryo sacs than mutant embryo sacs are positive for *DR5*.

**Table 3:** **Expression of *PIN1* and *DR5* reporters in embryo sacs of plants heterozygous for *Lxm1* and hemizygous for the transgene**.

	**Wild type without fluorescent reporter expression**	**Wild type with fluorescent reporter expression**	***Lxm1* without fluorescent reporter expression**	***Lxm1* with fluorescent reporter expression**
*Lxm1-O/+ pPIN1::PIN1-YFP/–*	16	16	13	0
*Lxm1-O/+ pDR5::RFP/–*	51	34	17	1^*^

* intermediate phenotype, either mild mutant phenotype or wild-type with smaller antipodal cluster.

The effect of a small antipodal cell cluster on *DR5* expression in the surrounding nucellar cells was also examined in the *Lxm1* mutant. A comparison was made between ovules which surrounded either normal or mutant embryo sacs, from the same heterozygous *Lxm1/+* mutant. The settings of the microscope needed to detect *DR5* expression were used as a proxy for the relative expression level of *DR5* in these ovules. Single mid-plane images were collected at three different gain settings to reduce the effects of photo-bleaching and each ovule was evaluated for the lowest setting at which *DR5* expression could be detected. *Lxm1* has a quantitative effect on the expression of *DR5* in the nucellar cells surrounding the antipodal cell cluster (Figure 7E). A higher gain is necessary to detect *DR5* in the nucellar cells around the small antipodal cell clusters of *Lxm1* mutant embryo sacs than around those of wild-type embryo sacs, indicating that *DR5* has lower expression around mutant embryo sacs than wild-type embryo sacs despite the fact that these two sets of nucellar cells are from ovules of the same plant and are therefore genetically identical.

## Discussion

Little is known about angiosperm antipodal cell function and development. The only evidence for antipodal cell function is based on implications from ultrastructural data (Diboll, 1968). The antipodal cells have many features that distinguish them from their sibling embryo sac cells, including: nuclear morphology, sucrose synthase activity, and cell wall invaginations. Some genetic evidence is also available for regulation of antipodal cell development including the influence of the central cell on antipodal cell persistence in Arabidopsis (Kagi et al., 2010) and of the egg cell on antipodal cell identity in maize (Krohn et al., 2012). Auxin signaling has been shown to occur in the maize antipodal cells (Lituiev et al., 2013), but no function for auxin in the embryo sac of maize was shown.

Localization of PIN1a protein suggests that auxin is transported away from the central cell and often toward the chalazal tip of the cluster, where the highest expression of *DR5* is located. Lower levels of DR5 expression are found throughout the antipodal cells and an even lower level in the surrounding nucellar cells. Indeed, PIN1a localization suggests that auxin is also transported from the antipodal cells into the surrounding cell layers. Analysis of antipodal cell morphology and *DR5* and *PIN1a* expression patterns reveals a dynamic pattern within the antipodal cell cluster. The chalazal-most antipodal cell of the cluster is unique, with unusual morphology and absence of *DR5* and *PIN1a* expression early in antipodal cell development and the highest level of *DR5* expression late. Several patterns of *PIN1a* expression were also seen in multicellular, mature or nearly mature, antipodal cell clusters. Whether or not these different patterns represent a development progression within antipodal cell clusters that are morphologically similar is unclear.

One hypothesis for regulation of embryo sac development is an antagonistic relationship between auxin and cytokinin with an inverse correlation between *DR5* and *TCS* expression as occurs during development of the embryonic root pole (Muller and Sheen, 2008). Interaction between auxin and cytokinin has been shown to act in root development (Moubayidin et al., 2009) shoot apical meristem development (Lee et al., 2009) and shoot branching (Shimizu-Sato et al., 2009) However, neither *DR5* nor *TCS* are expressed in the micropylar or central domains of the embryo sac (e.g., the central cell or the egg apparatus), suggesting that embryo sac cell identity is not regulated by antagonism of auxin and cytokinin signaling within the embryo sac. However, *TCS* expression was detected in the nucellus surrounding the antipodal cells, a region that also has weak auxin signaling as revealed by *DR5* expression. It may be that interplay between cytokinin and auxin in these cells is important to prevent proliferation of the nucellus next to the growing embryo sac. Promotion of cell division by auxin in the antipodal cells and antagonism of this action by cytokinin would be similar to the effects of auxin and cytokinin in promoting and inhibiting, respectively, the early divisions that establish lateral root primordia (Laplaze et al., 2007). The overlap of *TCS* expression with the lower expression of *DR5* in the nucellus around the antipodal cells and the absence of *TCS* from the antipodal cells that have higher expression of *DR5* is similar to the relationship between the expression of *TCS* and *DR5* in the embryonic root stem cell lineage with *TCS* and low *DR5* expression in the lenticular cell and *DR5* expression in the basal cell (Muller and Sheen, 2008).

In the mature maize embryo sac both *DR5* and *PIN1* are expressed strongly and specifically in the antipodal cell cluster, in stark contrast to Arabidopsis. This raises the possibility that the difference in auxin levels in the maize and Arabidopsis embryo sacs may be responsible for their different antipodal cell biology. One prediction based on these possibilities is that impaired auxin signaling or reduced auxin levels in the antipodal cells could disrupt their identity and/or proliferation. The dominant *Lxm1-O* mutation blocks proliferation of and auxin signaling in the antipodal cells. Despite failing to proliferate, *Lxm* mutant antipodal cells have normal morphology based on size, staining, and fluorescent properties. The phenotype of *Lxm1* mutants supports a model in which auxin promotes antipodal cell growth rather than antipodal cell identity. *Lxm1-O* plants also have defects in leaf polarity, leaf primordia size, and flowering time (Schichnes et al., 1997; Schichnes and Freeling, 1998). It has yet to be determined if these other *Lxm1-O* phenotypes are associated with defects in auxin signaling, but all of these processes are impacted by auxin (Reinhardt et al., 2000; Ellis et al., 2005; Okushima et al., 2005; Garcia et al., 2006; Richter et al., 2013; Qi et al., 2014).

The nucellar cells of *Lxm1-O/+* ovules surrounding wild-type embryo sacs express *DR5* normally, but those surrounding mutant embryo sacs do not. This is consistent with a model in which the nucellar *DR5* expression depends on the antipodal cell cluster rather than being cell-autonomous or dependent on other nucellar cells. Under this model, the *Lxm1-O* mutation would interfere with auxin signaling in the nucellus by reducing the source of auxin for these cells from the antipodals. The rate-limiting step for auxin synthesis is performed by the *YUCCA* class of flavin monooxygenases (Dai et al., 2013). Maize genes in *YUC10* and *YUC2/6* branches of this family show higher expression in the embryo sac enriched samples than the surrounding ovule tissue, consistent with local auxin synthesis, perhaps in the antipodal cells themselves.

Since the antipodal cells persist after fertilization in maize and other grasses (Randolph, 1936; Engell, 1994), they may even act as a transfer tissue for early seed development. One prediction for this model is that a reduction of antipodal cell transfer ability, possibly by reducing cluster size, would reduce growth of the seed and/or embryo sac. The maize *stunter1* mutant causes a reduction in antipodal cell number, central cell size, and seed size, but also causes a reduction in early stage female gametophytes before cellularization, suggesting that the *stt1* gene product is involved in growth of the embryo sac and antipodal cells independently of each other rather than a reduced antipodal cell cluster causing embryo sac and seed size reduction (Phillips and Evans, 2011). Consequently, the effects of *Lxm1* on antipodal cell development and seed size were also analyzed. Reciprocal crosses between *Lxm1* and wild type revealed that mutant females produce small kernels at a greater severity and frequency than mutant males.

The causes of the miniature kernel phenotype of *Lxm* mutants are potentially complex, however. The fact that the miniature kernel phenotype correlates with inheritance of the *Lxm* mutation through the embryo sac demonstrates that this miniature phenotype is not an incompletely penetrant maternal sporophyte effect (e.g., variable ovule morphology causing some seeds to develop abnormally), but rather a consequence of the genotype of the embryo sac or the endosperm. Seed growth may be sensitive to dosage of the dominant *Lxm* mutations in the endosperm rather than being a consequence of fertilization of abnormal embryo sacs. This is consistent with the milder effect on seed size when using *Lxm* as a male than a female since there would be only one copy of *Lxm1-O* in the endosperm compared to two copies of *Lxm1-O* in the endosperm when used as a female parent. However, it is also possible that the stronger effect of *Lxm* through the female is a combination of a post-fertilization effect of *Lxm* in the endosperm plus a maternal effect of *Lxm*, potentially because of the abnormal antipodal cells. To determine if the antipodal cells play a role in seed size, additional experiments are necessary, such as the targeted ablation of the antipodal cells or analysis of less pleiotropic mutants to determine if antipodal cell defects cause maternal gametophyte effects on seed development.

The antipodal cell growth pattern that is widespread in the grasses suggests that the antipodal cells serve a common function in the grasses. The presence of cell wall invaginations on the sides of antipodal cells facing the nucellus (Diboll, 1968) suggests that the antipodal cell cluster functions as a transfer tissue for the embryo sac. Auxin may function to promote the growth of the antipodal cells for this function. In contrast, the lack proliferation and auxin signaling in these cells may correlate with the lack of this function in Arabidopsis. However, as in maize, the antipodal cells of Arabidopsis can persist through embryo sac development and after fertilization (Randolph, 1936; Song et al., 2014). Perhaps the antipodal cells serve as a signaling center providing positional information for the embryo sac or developing endosperm in maize and Arabidopsis, while the transfer tissue function has a more limited distribution.

### Conflict of interest statement

The authors declare that the research was conducted in the absence of any commercial or financial relationships that could be construed as a potential conflict of interest.
