# Supplementary material for: Correlation between a loss of auxin signaling and a loss of proliferation in maize antipodal cells
Source: Front Plant Sci. 2015 Mar 26;6:187. doi: 10.3389/fpls.2015.00187 (PMC4374392; doi:10.3389/fpls.2015.00187)
Supplement: Supplementary file 1 [file Table1.DOCX]

Supplemental Tables.

| Table S1. Expression of *PIN* genes in maize gametophytes | | | | | |
| --- | --- | --- | --- | --- | --- |
|  |  | Embryo-sac-enriched | Ovule with embryo sac removed | Seedling shoot | Mature Pollen |
| GRMZM2G098643 | ZmPIN1a | 0.364 | 0.006 | 1.106 | 0.000 |
| GRMZM2G171702 | ZmPIN1d | 2.786 | 0.271 | 8.483 | 0.016 |
| GRMZM5G839411 | ZmPIN8 | 0.338 | 0.052 | 1.057 | 0.000 |
| GRMZM2G126260 | ZmPIN10a | 6.958 | 1.832 | 2.620 | 0.187 |
| GRMZM2G160496 | ZmPIN10b | 0.058 | 0.000 | 0.000 | 0.000 |
| GRMZM2G025742 | ZmPIN5a | 0.000 | 0.000 | 1.659 | 0.000 |
| GRMZM2G040911 | ZmPIN5c | 0.000 | 0.000 | 0.207 | 0.000 |
| GRMZM2G149184 | ZmPIN1c | 0.015 | 0.019 | 1.305 | 0.000 |
| GRMZM5G859099 | ZmPIN9 | 0.085 | 0.110 | 0.458 | 0.000 |
| GRMZM2G074267 | ZmPIN1b | 0.086 | 0.312 | 18.005 | 0.000 |
| Expression values are given in Fragments per Kilobase per Million reads (FPKM). Genes are sorted highest to lowest by ratio of Embryo sac to surrounding Ovule expression. Genes up-regulated two-fold in the embryo-sac-enriched samples (and over 0.1 FPKM) compared to the surrounding ovule tissue are indicated in red, while genes with higher expression in the surrounding ovule tissue than the embryo sac are indicated in blue. Genes indicated in orange have higher expression in the embryo sac than the surrounding ovule but either fall below the 0.1 FPKM cutoff or are only 1.5 to 2.0 fold higher in the embryo sac compared to the ovule. (ZmPIN2 is not included because it is not part of the genome assembly.) | | | | | |

| Table S2. Expression of *AUX1-like* genes in maize gametophytes | | | | |
| --- | --- | --- | --- | --- |
|  | Embryo-sac-enriched | Ovule with embryo sac removed | Seedling shoot | Mature Pollen |
| GRMZM2G129413 | 1.395 | 0.166 | 21.391 | 0.000 |
| GRMZM2G149481 | 0.095 | 0.015 | 4.814 | 0.014 |
| GRMZM2G127949 | 7.224 | 3.870 | 2.648 | 1.331 |
| GRMZM2G045057 | 0.000 | 0.040 | 2.616 | 0.000 |
| GRMZM2G067022 | 0.037 | 0.146 | 1.086 | 0.028 |
| Expression values are given in FPKM. Genes are sorted highest to lowest by ratio of Embryo sac to surrounding Ovule expression. Genes up-regulated two-fold in the embryo-sac-enriched samples (and over 0.1 FPKM) compared to the surrounding ovule tissue are indicated in red, while genes with higher expression in the surrounding ovule tissue than the embryo sac are indicated in blue. Genes indicated in orange have higher expression in the embryo sac than the surrounding ovule but either fall below the 0.1 FPKM cutoff or are only 1.5 to 2.0 fold higher in the embryo sac compared to the ovule. | | | | |

| Table S3. Expression of *brachytic2-like* ABC transporter genes in maize gametophytes | | | | |
| --- | --- | --- | --- | --- |
|  | Embryo-sac-enriched | Ovule with embryo sac removed | Seedling shoot | Mature Pollen |
| GRMZM2G004748 | 2.374 | 0.674 | 7.027 | 0.038 |
| GRMZM2G441722 | 1.502 | 0.642 | 0.135 | 0.022 |
| brachytic2 (br2) GRMZM2G315375 | 1.098 | 0.475 | 3.986 | 0.000 |
| GRMZM2G125424 | 19.003 | 10.841 | 3.586 | 0.120 |
| GRMZM2G072850 | 0.632 | 0.427 | 0.335 | 0.074 |
| GRMZM5G891159 | 5.147 | 5.637 | 3.044 | 0.007 |
| GRMZM2G085236 | 0.043 | 0.120 | 1.056 | 0.004 |
| GRMZM2G333183 | 0.248 | 0.721 | 0.233 | 0.008 |
| Expression values are given in FPKM. Genes are sorted highest to lowest by ratio of Embryo sac to surrounding Ovule expression. Genes up-regulated two-fold in the embryo-sac-enriched samples (and over 0.1 FPKM) compared to the surrounding ovule tissue are indicated in red, while genes with higher expression in the surrounding ovule tissue than the embryo sac are indicated in blue. Genes indicated in orange have higher expression in the embryo sac than the surrounding ovule but either fall below the 0.1 FPKM cutoff or are only 1.5 to 2.0 fold higher in the embryo sac compared to the ovule. | | | | |

| Table S4. Expression of *PID/WAG* protein kinase genes in maize gametophytes | | | | |
| --- | --- | --- | --- | --- |
|  | Embryo-sac-enriched | Ovule with embryo sac removed | Seedling shoot | Mature Pollen |
| GRMZM2G103559 | 6.020 | 0.199 | 2.021 | 0.000 |
| GRMZM2G019567 | 0.115 | 0.020 | 3.433 | 0.000 |
| GRMZM2G037386 | 0.062 | 0.058 | 2.443 | 0.000 |
| GRMZM2G171822 | 0.000 | 0.008 | 0.061 | 0.000 |
| GRMZM2G466897 | 0.000 | 0.000 | 0.000 | 0.000 |
| Expression values are given in FPKM. Genes are sorted highest to lowest by ratio of Embryo sac to surrounding Ovule expression. Genes up-regulated two-fold in the embryo-sac-enriched samples (and over 0.1 FPKM) compared to the surrounding ovule tissue are indicated in red, while genes with higher expression in the surrounding ovule tissue than the embryo sac are indicated in blue. Genes indicated in orange have higher expression in the embryo sac than the surrounding ovule but either fall below the 0.1 FPKM cutoff or are only 1.5 to 2.0 fold higher in the embryo sac compared to the ovule. | | | | |

| Table S5. Expression of *TAA-like* genes in maize gametophytes | | | | |
| --- | --- | --- | --- | --- |
|  | Embryo-sac-enriched | Ovule with embryo sac removed | Seedling shoot | Mature Pollen |
| GRMZM2G141810 | 2.477 | 0.000 | 0.052 | 0.000 |
| GRMZM2G127160 | 4.225 | 0.007 | 0.016 | 2.048 |
| GRMZM2G066345 | 3.397 | 0.045 | 0.912 | 0.090 |
| GRMZM2G054115 | 2.198 | 0.365 | 6.966 | 0.258 |
| GRMZM2G127308 | 0.601 | 0.394 | 2.538 | 0.024 |
| GRMZM2G181135 | 0.000 | 0.000 | 0.358 | 0.288 |
| Expression values are given in Fragments per Kilobase per Million reads (FPKM). Genes are sorted highest to lowest by ratio of Embryo sac to surrounding Ovule expression. Genes up-regulated two-fold in the embryo-sac-enriched samples (and over 0.1 FPKM) compared to the surrounding ovule tissue are indicated in red, while genes with higher expression in the surrounding ovule tissue than the embryo sac are indicated in blue. Genes indicated in orange have higher expression in the embryo sac than the surrounding ovule but either fall below the 0.1 FPKM cutoff or are only 1.5 to 2.0 fold higher in the embryo sac compared to the ovule. | | | | |

| Table S6. Expression of *YUCCA1-like* genes in maize gametophytes | | | | | |
| --- | --- | --- | --- | --- | --- |
|  |  | Embryo-sac-enriched | Ovule with embryo sac removed | Seedling shoot | Mature Pollen |
| GRMZM2G107761 | ZmYUC10a | 9.449 | 0.049 | 0.000 | 0.169 |
| GRMZM2G013045 | ZmYUC6a | 1.311 | 0.175 | 0.111 | 0.000 |
| GRMZM2G091819 | ZmYUC10b | 0.370 | 0.053 | 0.000 | 1.862 |
| GRMZM2G328780 | ZmYUC6b | 1.411 | 0.268 | 0.000 | 0.467 |
| GRMZM2G017193 | ZmYUC2 | 1.021 | 0.396 | 1.040 | 0.287 |
| GRMZM2G019515 | ZmYUC10c | 0.078 | 0.067 | 9.661 | 0.000 |
| GRMZM2G141383 | ZmYUC3a | 0.000 | 0.000 | 0.855 | 0.000 |
| GRMZM2G333478 | ZmYUC3c | 0.000 | 0.000 | 0.656 | 0.000 |
| GRMZM2G159393 | ZmYUC3b | 0.000 | 0.000 | 0.197 | 0.000 |
| GRMZM2G480386 | ZmYUC1 | 0.000 | 0.000 | 0.000 | 0.000 |
| GRMZM2G132489 | ZmYUC3d | 0.304 | 0.901 | 2.122 | 0.000 |
| Expression values are given in FPKM. Genes are sorted highest to lowest by ratio of Embryo sac to surrounding Ovule expression. Genes up-regulated two-fold in the embryo-sac-enriched samples (and over 0.1 FPKM) compared to the surrounding ovule tissue are indicated in red, while genes with higher expression in the surrounding ovule tissue than the embryo sac are indicated in blue. Genes indicated in orange have higher expression in the embryo sac than the surrounding ovule but either fall below the 0.1 FPKM cutoff or are only 1.5 to 2.0 fold higher in the embryo sac compared to the ovule. | | | | | |

| Table S7. Expression of *TIR1-like* genes in maize gametophytes | | | | |
| --- | --- | --- | --- | --- |
|  | Embryo-sac-enriched | Ovule with embryo sac removed | Seedling shoot | Mature Pollen |
| GRMZM2G024180 | 14.217 | 7.193 | 2.546 | 0.240 |
| GRMZM2G135978 | 9.548 | 6.269 | 24.269 | 0.015 |
| GRMZM2G095786 | 2.747 | 2.023 | 3.405 | 0.000 |
| GRMZM2G427529 | 9.232 | 7.763 | 6.328 | 0.287 |
| GRMZM5G848945 | 23.584 | 21.637 | 26.165 | 0.030 |
| GRMZM2G398848 | 3.190 | 3.508 | 2.440 | 0.004 |
| GRMZM2G137451 | 8.490 | 12.385 | 10.431 | 0.008 |
| GRMZM2G155849 | 14.277 | 25.638 | 9.147 | 0.850 |
| Expression values are given in FPKM. Genes are sorted highest to lowest by ratio of Embryo sac to surrounding Ovule expression. Genes up-regulated two-fold in the embryo-sac-enriched samples (and over 0.1 FPKM) compared to the surrounding ovule tissue are indicated in red, while genes with higher expression in the surrounding ovule tissue than the embryo sac are indicated in blue. Genes indicated in orange have higher expression in the embryo sac than the surrounding ovule but either fall below the 0.1 FPKM cutoff or are only 1.5 to 2.0 fold higher in the embryo sac compared to the ovule. | | | | |

| Table S8. Expression of *ARF* genes in maize gametophytes | | | | | |
| --- | --- | --- | --- | --- | --- |
|  |  | Embryo-sac-enriched | Ovule with embryo sac removed | Seedling shoot | Mature Pollen |
| GRMZM5G808366 | ZmARF5 | 0.210 | 0.024 | 0.000 | 0.000 |
| GRMZM2G081406 | ZmARF15 | 13.307 | 2.505 | 3.790 | 0.000 |
| GRMZM2G390641 | ZmARF21 | 1.773 | 0.362 | 6.021 | 0.011 |
| GRMZM2G102845 | ZmARF20 | 12.334 | 3.808 | 3.215 | 0.062 |
| GRMZM2G153233 | ZmARF2 | 30.493 | 11.971 | 9.113 | 0.021 |
| GRMZM2G159399 | ZmARF17 | 41.135 | 16.961 | 7.106 | 0.020 |
| GRMZM2G023813 | ZmARF31 | 0.085 | 0.040 | 0.000 | 0.061 |
| AC207656.3_FG002 | ZmARF19 | 0.298 | 0.145 | 1.505 | 0.777 |
| GRMZM2G317900 | ZmARF35 | 16.709 | 8.192 | 3.979 | 0.008 |
| GRMZM2G056120 | ZmARF11 | 1.831 | 0.966 | 5.127 | 0.000 |
| GRMZM2G005284 | ZmARF38 | 0.609 | 0.404 | 0.000 | 0.250 |
| GRMZM2G028980 | ZmARF16 | 4.336 | 2.929 | 3.707 | 0.418 |
| GRMZM2G352159 | ZmARF8 | 12.211 | 8.495 | 1.996 | 0.498 |
| GRMZM2G338259 | ZmARF10 | 41.411 | 31.147 | 17.368 | 0.013 |
| GRMZM2G437460 | ZmARF12 | 4.476 | 3.408 | 8.117 | 0.013 |
| GRMZM2G034840 | ZmARF4 | 0.434 | 0.344 | 4.074 | 0.000 |
| GRMZM2G169820 | ZmARF1 | 3.824 | 3.450 | 13.718 | 0.000 |
| GRMZM2G378580 | ZmARF13 | 14.693 | 13.646 | 12.936 | 0.003 |
| GRMZM2G702026 | ZmARF36 | 11.506 | 11.610 | 24.040 | 0.007 |
| GRMZM2G030710 | ZmARF24 | 4.921 | 5.078 | 15.117 | 0.008 |
| GRMZM2G006042 | ZmARF28 | 30.765 | 31.832 | 3.287 | 0.000 |
| GRMZM2G116557 | ZmARF25 | 18.434 | 19.247 | 8.079 | 0.010 |
| GRMZM2G137413 | ZmARF14 | 2.084 | 2.501 | 2.454 | 0.000 |
| GRMZM2G475263 | ZmARF7 | 0.886 | 1.083 | 3.635 | 0.008 |
| GRMZM2G035405 | ZmARF18 | 13.104 | 18.173 | 26.545 | 0.003 |
| GRMZM2G160005 | ZmARF27 | 5.207 | 7.835 | 0.468 | 0.379 |
| GRMZM2G073750 | ZmARF9 | 5.083 | 7.891 | 8.199 | 0.013 |
| GRMZM2G078274 | ZmARF3 | 37.402 | 60.198 | 51.678 | 0.000 |
| GRMZM2G181254 | ZmARF32 | 0.100 | 0.164 | 0.154 | 0.000 |
| GRMZM2G086949 | ZmARF29 | 0.649 | 1.095 | 1.975 | 0.000 |
| GRMZM2G081158 | ZmARF34 | 3.763 | 6.948 | 9.452 | 0.000 |
| GRMZM2G441325 | ZmARF23 | 0.505 | 0.951 | 6.711 | 0.026 |
| GRMZM2G179121 | ZmARF33 | 0.000 | 0.000 | 0.000 | 0.000 |
| GRMZM2G122614 | ZmARF6 | 0.000 | 0.000 | 0.065 | 0.011 |
| GRMZM5G874163 | ZmARF26 | 0.291 | 0.628 | 3.762 | 0.000 |
| Expression values are given in FPKM. Genes are sorted highest to lowest by ratio of Embryo sac to surrounding Ovule expression. Genes up-regulated two-fold in the embryo-sac-enriched samples (and over 0.1 FPKM) compared to the surrounding ovule tissue are indicated in red, while genes with higher expression in the surrounding ovule tissue than the embryo sac are indicated in blue. Genes indicated in orange have higher expression in the embryo sac than the surrounding ovule but either fall below the 0.1 FPKM cutoff or are only 1.5 to 2.0 fold higher in the embryo sac compared to the ovule. | | | | | |

| Table S9. Expression of *IAA* genes in maize gametophytes | | | | | |
| --- | --- | --- | --- | --- | --- |
|  |  | Embryo-sac-enriched | Ovule with embryo sac removed | Seedling shoot | Mature Pollen |
| GRMZM2G000158 | ZmIAA18 | 1.395 | 0.021 | 2.156 | 0.000 |
| GRMZM2G366373 |  | 1.013 | 0.053 | 5.303 | 5.278 |
| GRMZM5G825707 |  | 1.765 | 0.099 | 0.467 | 0.000 |
| GRMZM2G031615 |  | 0.342 | 0.019 | 3.458 | 0.000 |
| GRMZM5G864847 |  | 3.878 | 0.332 | 1.566 | 0.000 |
| GRMZM2G057067 | ZmIAA9 | 4.108 | 0.583 | 23.717 | 0.008 |
| GRMZM2G128421 | ZmIAA15 | 4.168 | 0.611 | 12.305 | 0.073 |
| GRMZM2G077356 | ZmIAA14 | 14.822 | 2.235 | 17.967 | 0.046 |
| GRMZM5G809195 |  | 1.015 | 0.225 | 11.524 | 12.683 |
| GRMZM2G104176 | ZmIAA4 | 9.317 | 2.190 | 0.787 | 0.008 |
| GRMZM2G167794 | ZmIAA8 | 17.265 | 4.922 | 27.261 | 0.000 |
| GRMZM2G084993 |  | 2.742 | 0.807 | 2.553 | 0.000 |
| GRMZM2G573324 |  | 2.219 | 0.675 | 16.911 | 0.000 |
| GRMZM2G079957 | ZmIAA1 | 15.446 | 5.550 | 11.012 | 0.079 |
| GRMZM2G130953 | ZmIAA27 | 1.958 | 0.851 | 8.266 | 0.040 |
| GRMZM2G035465 | ZmIAA28 | 26.937 | 13.413 | 38.424 | 0.049 |
| GRMZM2G004696 | ZmIAA5 | 9.722 | 4.976 | 62.267 | 0.016 |
| GRMZM2G030465 | ZmIAA17 | 0.101 | 0.054 | 0.151 | 0.000 |
| GRMZM2G159285 | ZmIAA2 | 2.809 | 1.602 | 31.745 | 0.056 |
| GRMZM2G163848 | ZmIAA29 | 12.407 | 7.166 | 34.478 | 0.149 |
| GRMZM2G359924 |  | 0.903 | 0.541 | 0.039 | 0.000 |
| GRMZM2G479834 |  | 0.105 | 0.064 | 0.021 | 0.000 |
| GRMZM2G147243 | ZmIAA21 | 47.093 | 30.059 | 191.738 | 0.077 |
| GRMZM2G152796 | ZmIAA13 | 0.080 | 0.054 | 9.379 | 0.055 |
| GRMZM2G115357 | ZmIAA25 | 405.923 | 274.404 | 28.124 | 0.068 |
| GRMZM2G037368 | ZmIAA10 | 7.523 | 5.144 | 35.027 | 0.090 |
| GRMZM2G142768 | ZmIAA12 | 5.136 | 3.556 | 10.623 | 0.047 |
| GRMZM2G138268 | ZmIAA7 | 0.590 | 0.615 | 29.861 | 0.000 |
| GRMZM2G121309 | ZmIAA16 | 0.305 | 0.369 | 6.135 | 0.000 |
| GRMZM2G074742 | ZmIAA6 | 0.797 | 1.356 | 1.516 | 0.000 |
| GRMZM2G079200 | ZmIAA19 | 6.834 | 13.353 | 7.121 | 0.000 |
| GRMZM2G074427 | ZmIAA23 | 0.154 | 0.303 | 1.804 | 0.000 |
| GRMZM2G134517 | ZmIAA31 | 0.000 | 0.000 | 0.000 | 0.063 |
| GRMZM2G141205 | ZmIAA22 | 0.000 | 0.000 | 0.000 | 0.000 |
| GRMZM2G001799 | ZmIAA30 | 0.000 | 0.005 | 0.182 | 0.015 |
| GRMZM2G059544 | ZmIAA11 | 0.000 | 0.007 | 0.613 | 0.000 |
| GRMZM2G048131 | ZmIAA26 | 0.000 | 0.052 | 0.210 | 0.000 |
| Expression values are given in FPKM. Genes are sorted highest to lowest by ratio of Embryo sac to surrounding Ovule expression. Genes up-regulated two-fold in the embryo-sac-enriched samples (and over 0.1 FPKM) compared to the surrounding ovule tissue are indicated in red, while genes with higher expression in the surrounding ovule tissue than the embryo sac are indicated in blue. Genes indicated in orange have higher expression in the embryo sac than the surrounding ovule but either fall below the 0.1 FPKM cutoff or are only 1.5 to 2.0 fold higher in the embryo sac compared to the ovule. | | | | | |
