# Supplementary material for: Correlation between a loss of auxin signaling and a loss of proliferation in maize antipodal cells
Source: Front Plant Sci. 2015 Mar 26;6:187. doi: 10.3389/fpls.2015.00187 (PMC4374392; doi:10.3389/fpls.2015.00187)
Supplement: Supplementary file 2 [file Presentation1.PPTX]

## Slide 1
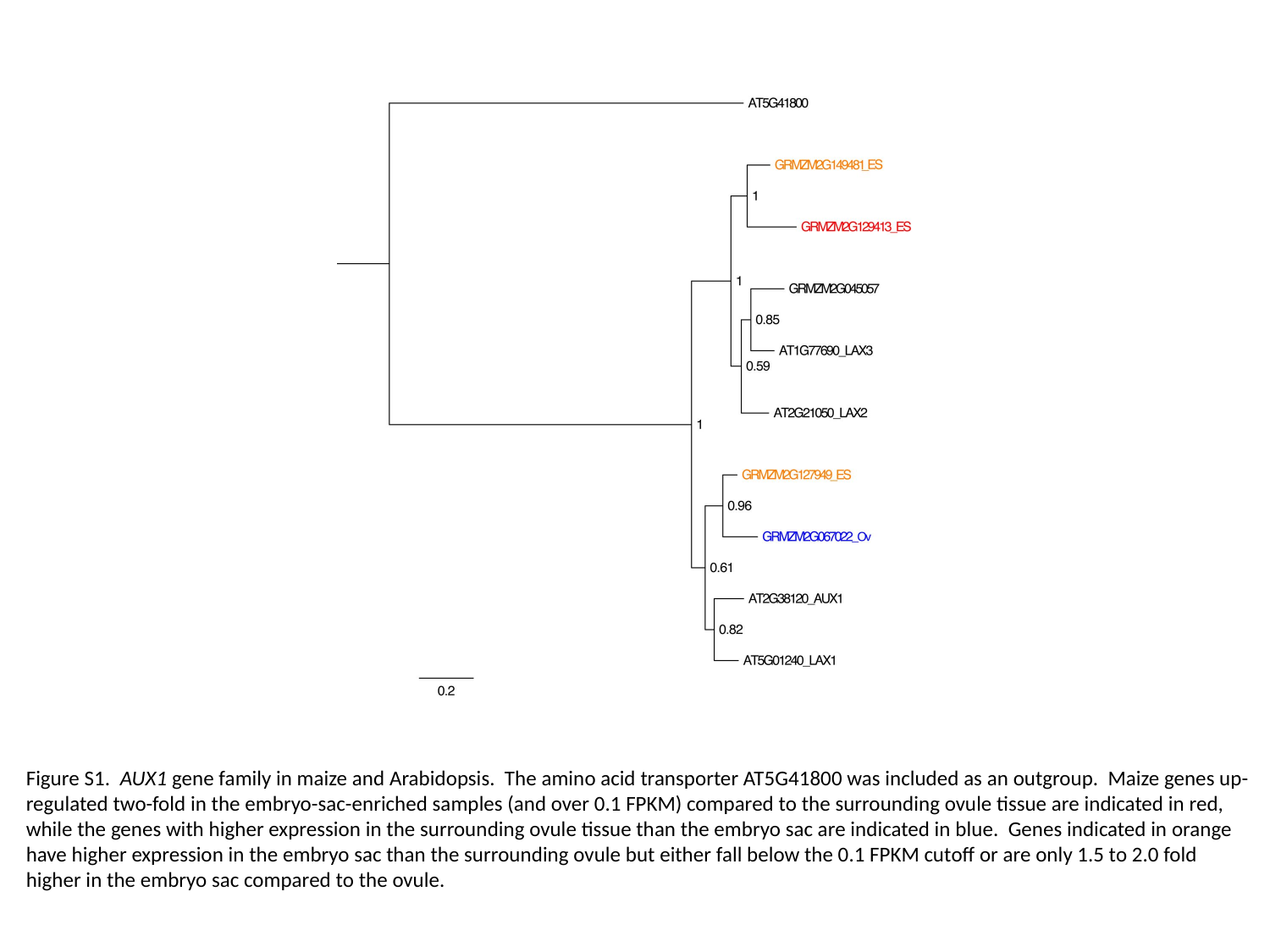

Figure S1. AUX1 gene family in maize and Arabidopsis. The amino acid transporter AT5G41800 was included as an outgroup. Maize genes up-regulated two-fold in the embryo-sac-enriched samples (and over 0.1 FPKM) compared to the surrounding ovule tissue are indicated in red, while the genes with higher expression in the surrounding ovule tissue than the embryo sac are indicated in blue. Genes indicated in orange have higher expression in the embryo sac than the surrounding ovule but either fall below the 0.1 FPKM cutoff or are only 1.5 to 2.0 fold higher in the embryo sac compared to the ovule.

## Slide 2
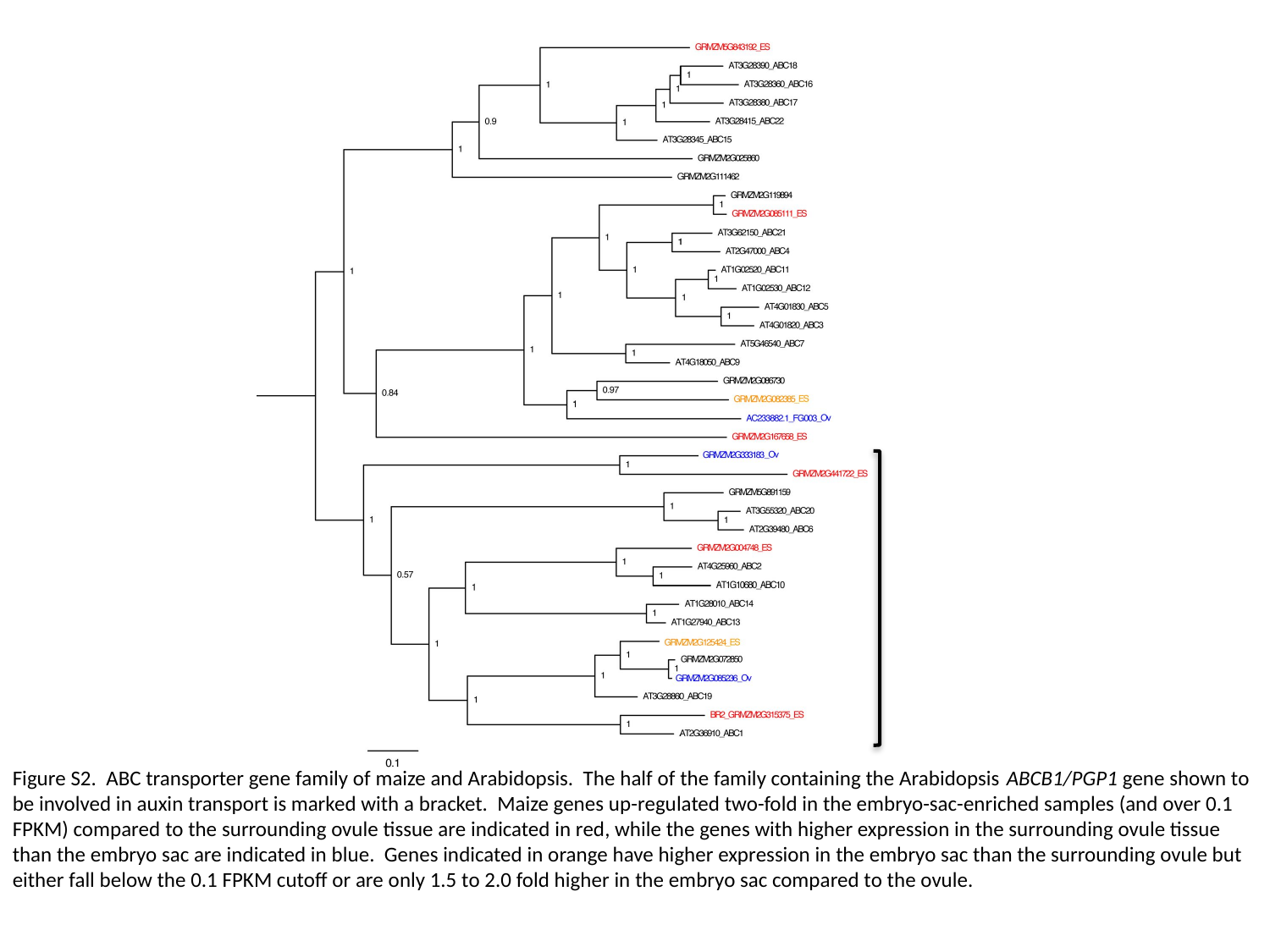

Figure S2. ABC transporter gene family of maize and Arabidopsis. The half of the family containing the Arabidopsis ABCB1/PGP1 gene shown to be involved in auxin transport is marked with a bracket. Maize genes up-regulated two-fold in the embryo-sac-enriched samples (and over 0.1 FPKM) compared to the surrounding ovule tissue are indicated in red, while the genes with higher expression in the surrounding ovule tissue than the embryo sac are indicated in blue. Genes indicated in orange have higher expression in the embryo sac than the surrounding ovule but either fall below the 0.1 FPKM cutoff or are only 1.5 to 2.0 fold higher in the embryo sac compared to the ovule.

## Slide 3
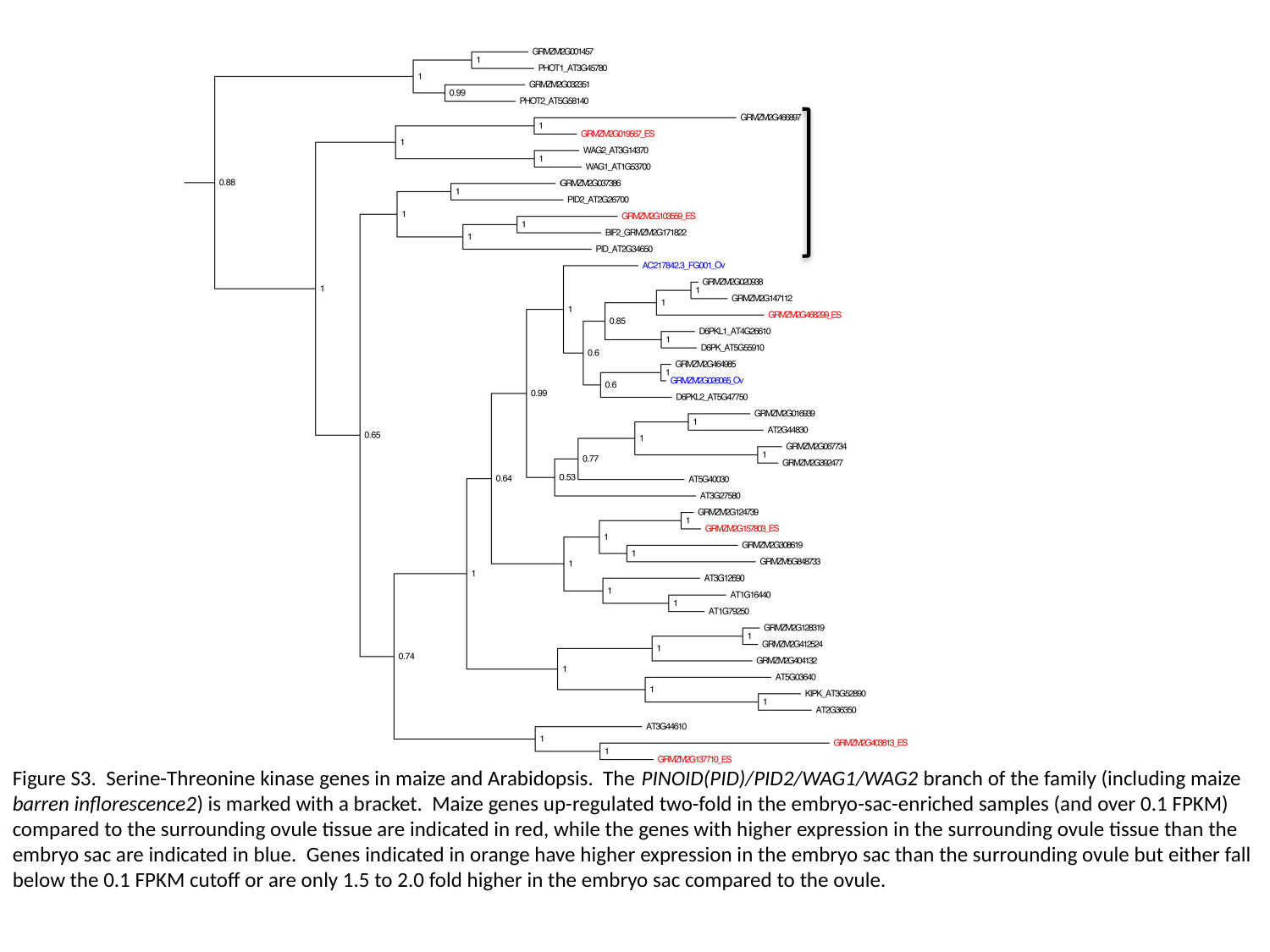

Figure S3. Serine-Threonine kinase genes in maize and Arabidopsis. The PINOID(PID)/PID2/WAG1/WAG2 branch of the family (including maize barren inflorescence2) is marked with a bracket. Maize genes up-regulated two-fold in the embryo-sac-enriched samples (and over 0.1 FPKM) compared to the surrounding ovule tissue are indicated in red, while the genes with higher expression in the surrounding ovule tissue than the embryo sac are indicated in blue. Genes indicated in orange have higher expression in the embryo sac than the surrounding ovule but either fall below the 0.1 FPKM cutoff or are only 1.5 to 2.0 fold higher in the embryo sac compared to the ovule.

## Slide 4
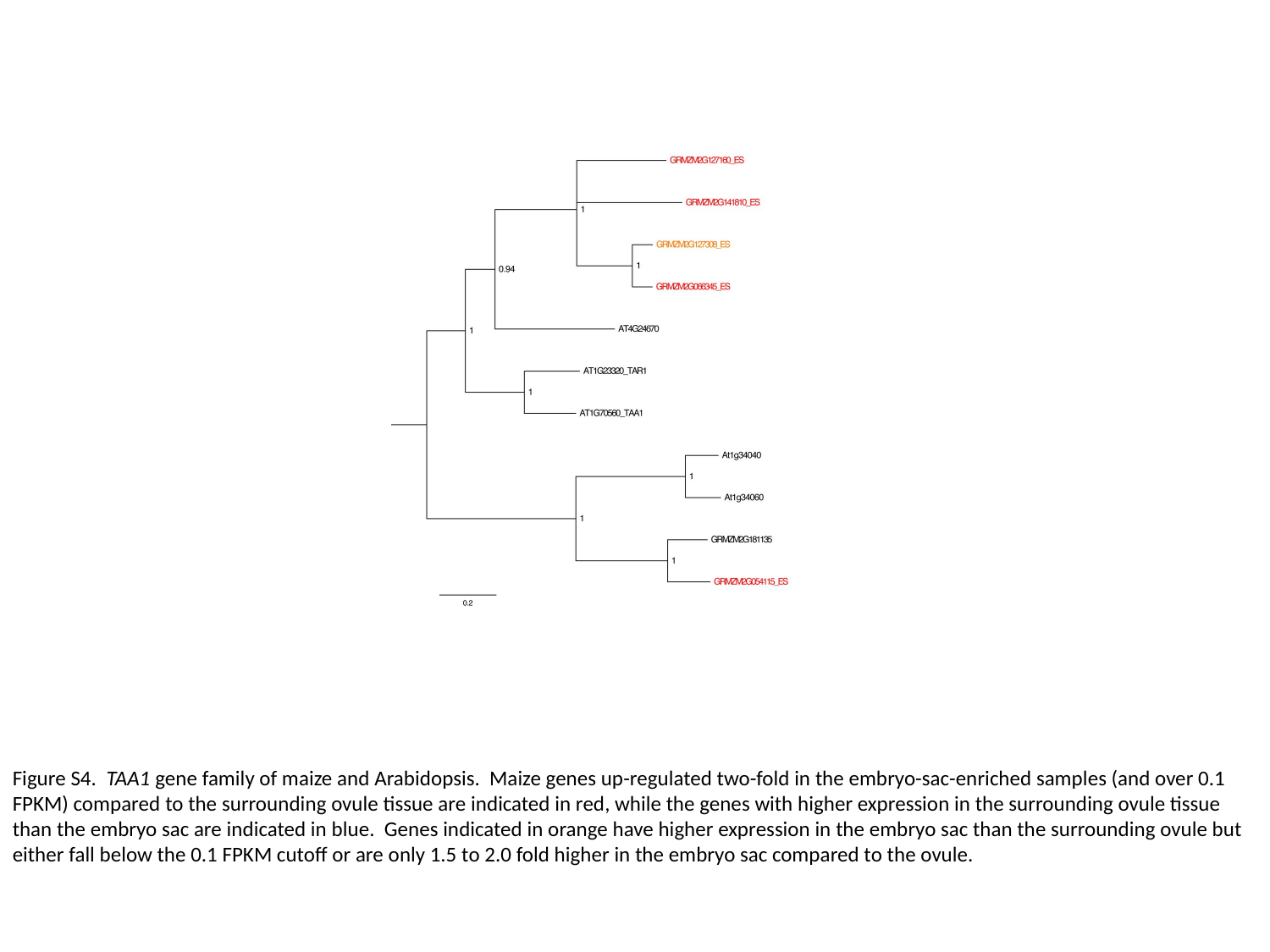

Figure S4. TAA1 gene family of maize and Arabidopsis. Maize genes up-regulated two-fold in the embryo-sac-enriched samples (and over 0.1 FPKM) compared to the surrounding ovule tissue are indicated in red, while the genes with higher expression in the surrounding ovule tissue than the embryo sac are indicated in blue. Genes indicated in orange have higher expression in the embryo sac than the surrounding ovule but either fall below the 0.1 FPKM cutoff or are only 1.5 to 2.0 fold higher in the embryo sac compared to the ovule.

## Slide 5
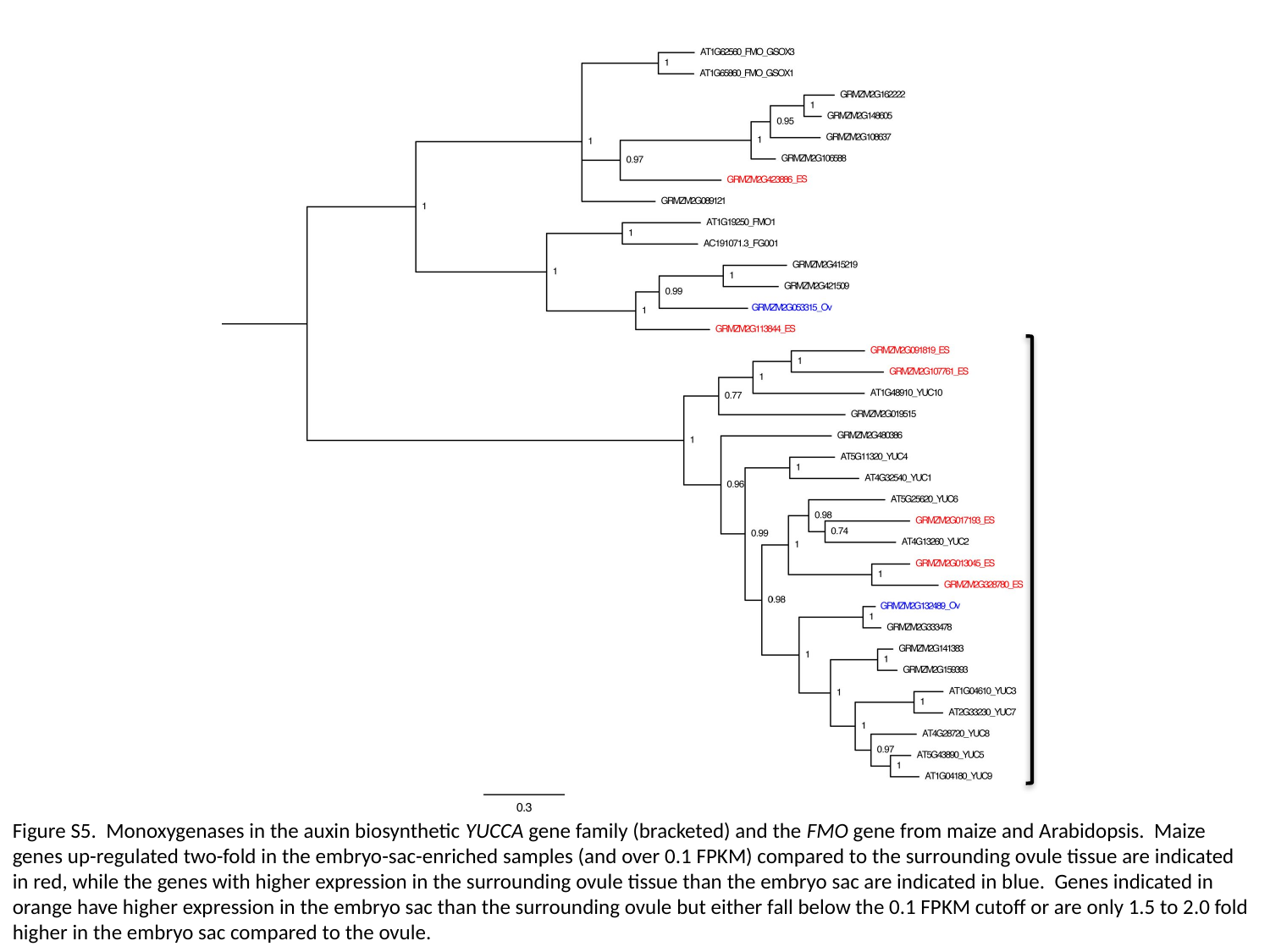

Figure S5. Monoxygenases in the auxin biosynthetic YUCCA gene family (bracketed) and the FMO gene from maize and Arabidopsis. Maize genes up-regulated two-fold in the embryo-sac-enriched samples (and over 0.1 FPKM) compared to the surrounding ovule tissue are indicated in red, while the genes with higher expression in the surrounding ovule tissue than the embryo sac are indicated in blue. Genes indicated in orange have higher expression in the embryo sac than the surrounding ovule but either fall below the 0.1 FPKM cutoff or are only 1.5 to 2.0 fold higher in the embryo sac compared to the ovule.

## Slide 6
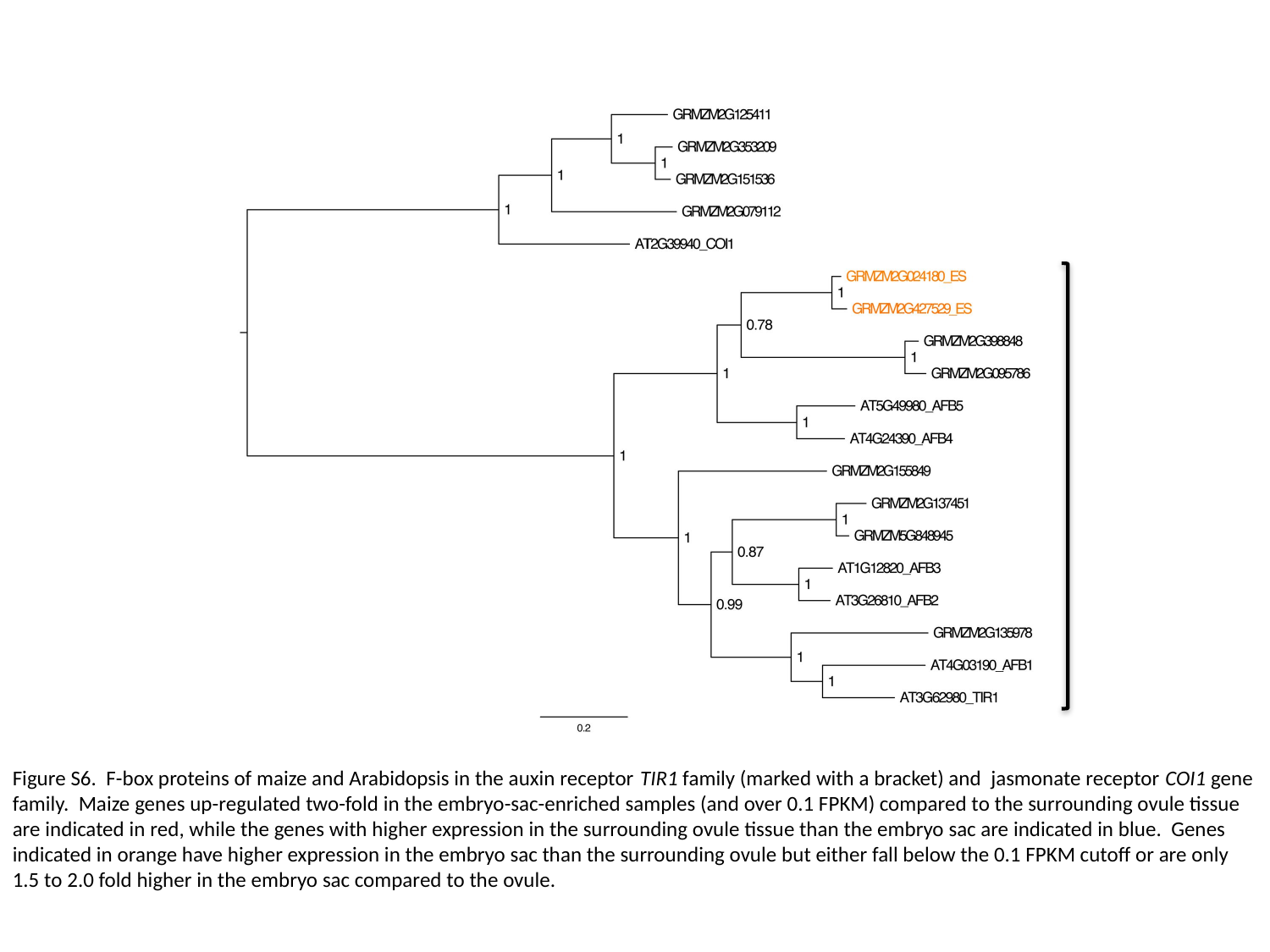

Figure S6. F-box proteins of maize and Arabidopsis in the auxin receptor TIR1 family (marked with a bracket) and jasmonate receptor COI1 gene family. Maize genes up-regulated two-fold in the embryo-sac-enriched samples (and over 0.1 FPKM) compared to the surrounding ovule tissue are indicated in red, while the genes with higher expression in the surrounding ovule tissue than the embryo sac are indicated in blue. Genes indicated in orange have higher expression in the embryo sac than the surrounding ovule but either fall below the 0.1 FPKM cutoff or are only 1.5 to 2.0 fold higher in the embryo sac compared to the ovule.

## Slide 7
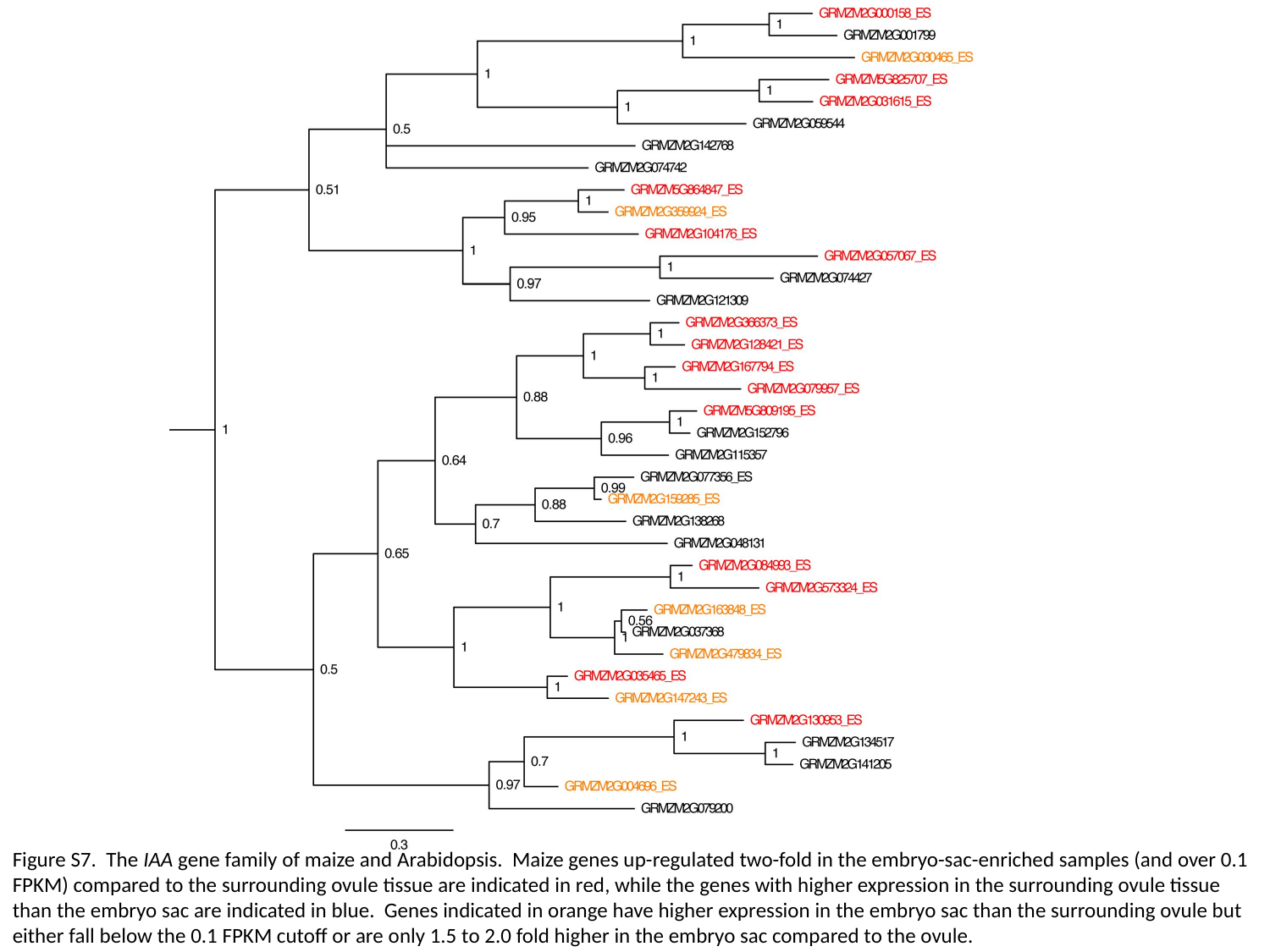

Figure S7. The IAA gene family of maize and Arabidopsis. Maize genes up-regulated two-fold in the embryo-sac-enriched samples (and over 0.1 FPKM) compared to the surrounding ovule tissue are indicated in red, while the genes with higher expression in the surrounding ovule tissue than the embryo sac are indicated in blue. Genes indicated in orange have higher expression in the embryo sac than the surrounding ovule but either fall below the 0.1 FPKM cutoff or are only 1.5 to 2.0 fold higher in the embryo sac compared to the ovule.
